# Molecular signatures reflecting microenvironmental metabolism and chemotherapy-induced immunogenic cell death in colorectal liver metastases

**DOI:** 10.18632/oncotarget.19350

**Published:** 2017-07-18

**Authors:** Olga Østrup, Vegar Johansen Dagenborg, Einar Andreas Rødland, Veronica Skarpeteig, Laxmi Silwal-Pandit, Krzysztof Grzyb, Audun Elnæs Berstad, Åsmund Avdem Fretland, Gunhild Mari Mælandsmo, Anne-Lise Børresen-Dale, Anne Hansen Ree, Bjørn Edwin, Vigdis Nygaard, Kjersti Flatmark

**Affiliations:** ^1^ Department of Tumor Biology, Institute for Cancer Research, Oslo University Hospital, The Norwegian Radium Hospital, Oslo, Norway; ^2^ Department of Cancer Genetics, Institute for Cancer Research, Oslo University Hospital, The Norwegian Radium Hospital, Oslo, Norway; ^3^ Department of Gastroenterological Surgery, Oslo University Hospital, The Norwegian Radium Hospital, Oslo, Norway; ^4^ Department of Hepato-Pancreato-Biliary Surgery, Oslo University Hospital, Rikshospitalet, Oslo, Norway; ^5^ Department of Pathology, Oslo University Hospital, Rikshospitalet, Oslo, Norway; ^6^ Department of Oncology, Akershus University Hospital, Lørenskog, Norway; ^7^ Department of Radiology, Oslo University Hospital, The Norwegian Radium Hospital, Oslo, Norway; ^8^ The Intervention Centre, Oslo University Hospital, Rikshospitalet, Oslo, Norway; ^9^ Institute of Clinical Medicine, Faculty of Medicine, University of Oslo, Oslo, Norway; ^10^ Department of Pharmacy, University of Tromsø - The Arctic University of Norway, Tromsø, Norway

**Keywords:** colorectal liver metastases, genomic profiling, neoadjuvant chemotherapy, immunogenic cell death

## Abstract

**Background:**

Metastatic colorectal cancer (CRC) is associated with highly variable clinical outcome and response to therapy. The recently identified consensus molecular subtypes (CMS1-4) have prognostic and therapeutic implications in primary CRC, but whether these subtypes are valid for metastatic disease is unclear. We performed multi-level analyses of resectable CRC liver metastases (CLM) to identify molecular characteristics of metastatic disease and evaluate the clinical relevance.

**Methods:**

In this ancillary study to the Oslo-CoMet trial, CLM and tumor-adjacent liver tissue from 46 patients were analyzed by profiling mutations (targeted sequencing), genome-wide copy number alteration (CNAs), and gene expression.

**Results:**

Somatic mutations and CNAs detected in CLM were similar to reported primary CRC profiles, while CNA profiles of eight metastatic pairs suggested intra-patient divergence. A CMS classifier tool applied to gene expression data, revealed the cohort to be highly enriched for CMS2. Hierarchical clustering of genes with highly variable expression identified two subgroups separated by high or low expression of 55 genes with immune-related and metabolic functions. Importantly, induction of genes and pathways associated with immunogenic cell death (ICD) was identified in metastases exposed to neoadjuvant chemotherapy (NACT).

**Conclusions:**

The uniform classification of CLM by CMS subtyping may indicate that novel class discovery approaches need to be explored to uncover clinically useful stratification of CLM. Detected gene expression signatures support the role of metabolism and chemotherapy in shaping the immune microenvironment of CLM. Furthermore, the results point to rational exploration of immune modulating strategies in CLM, particularly by exploiting NACT-induced ICD.

## INTRODUCTION

Extensive effort has been put into molecular classification of colorectal cancer (CRC), recently culminating in a consensus classification of the disease in four distinct consensus molecular subtypes (CMS1-4), which have implications for prognosis and treatment [1]. However, the discovery and definition of these subtypes is based on data originating from analysis of primary tumors, which may not fully capture the effects of metastatic progression. Metastases often retain fundamental genomic features of the primary tumor, exemplified by reports of high concordance between primary tumors and metastases regarding *KRAS*, *BRAF* and *PIK3CA* mutations [2]. However, molecular heterogeneity of the primary tumor may result in clonal selection during metastatic progression, and the metastatic phenotype is also subject to tumor stroma cross-talk and organ-specific microenvironmental influence. Therapy given throughout the disease course, including chemotherapy, radiotherapy and surgery, may also constitute environmental factors of importance for metastatic dissemination and growth. The potential changes in the metastatic phenotype may influence response to chemotherapy, development of therapy resistance, and ultimately disease outcome. Treatment of metastatic CRC remains a major challenge, and increased focus on the molecular biology of metastatic tumors is warranted in order to aim therapy at relevant targets.

Up to 50% of patients with CRC develop metastatic disease, which is associated with poor survival outcome, and the liver is the most common metastatic site [3]. Surgery is the only curative treatment option, but only 20% of patients with colorectal liver metastases (CLM) can be resected [4], and the risk of disease recurrence is high after surgical resection [5–7]. The Oslo-CoMet trial (*Oslo randomized laparoscopic vs open liver resection for colorectal liver metastases study*; NCT01516710) is the first randomized trial to compare laparoscopic and open resection of CLM (randomization of 280 cases completed February 2016) [8]. The study includes long-term oncologic outcome as a secondary endpoint, and the molecular analysis of CLM presented in this study is based on the first 71 included cases. The main aim of this ancillary study was to generate a broad molecular overview of resectable CLM, specifically to reveal key genomic determinants of molecular subgroups with potential translational value. Using a multilevel approach, we characterized CLM and elucidated transcriptomic changes associated with molecular and clinicopathological features. This is to our knowledge the first report identifying distinct immune-related signatures that may be exploited in novel strategies to optimize treatment outcomes in CLM, including those predicted to be classified as CMS2, a subgroup generally characterized by low expression of immune signatures.

## RESULTS

### Clinicopathological characteristics and outcome

The clinicopathological characteristics are sum-marized in Table 1. The study cohort (n=46) consisted of 27 (59%) men and 19 (41%) women. All primary tumors were adenocarcinomas and 39 (85%) cases were classified as moderately differentiated. The primary tumor was located in the right colon in 11 cases (24%), left colon in 16 (35%), and rectum in 19 (41%). Twenty-four patients (52%) had lymph node metastases in the primary tumor specimen, and 32 patients (70%) had synchronous liver metastases. Eight patients (17%) previously had one surgical procedure for CLM. One patient had potentially resectable lung metastases at the time of inclusion, and in one patient liver resection was performed in the interval between chemoradiotherapy and resection of the primary rectal tumor. Most patients had good performance status at the time of liver surgery, as assessed using the Eastern Cooperative Oncology Group (ECOG) performance status score, with 34 ECOG 0 (74%), 10 ECOG 1 (22%), and only 2 ECOG 2 (4%). Most patients had a low clinical risk score (CRS) [9]; 29 patients (76%) had a CRS of 0-2, and 9 (24%) scored 3-4, while none of the patients achieved the maximum score of 5. Following neoadjuvant chemotherapy (NACT), ten patients (67%) had stable disease, 3 patients (20%) had a partial response, and 2 patients (13%) had progressive disease. The median follow-up time was 42 months (6-51 months) from CoMet operation. At censoring, 18 of the 46 patients (39%) had died; 29 (63%) patients had developed disease recurrence, of which the first recurrence site was hepatic in 22 cases (76%).

**Table 1:** Clinicopathological characteristics of the study cohort

Variable		Number	%
Age		68	(45-81)
Gender			
	Male	27	59
	Female	19	41
Performance Status			
	ECOG 0	34	74
	ECOG 1	10	22
	ECOG 2	2	4
Primary tumor localization			
	Right Colon	11	24
	Left Colon	16	35
	Rectum	19	41
T-stage primary tumor			
	T1-T2	5	11
	T3	31	67
	T4	9	20
	NA	1	2
N-stage primary tumor			
	N0	22	48
	N+	24	52
Primary tumor histological differentiation			
	Well	3	7
	Moderate	39	85
	Poor	2	4
	NA	2	4
Liver metastases			
	Synchronous	32	70
	Metachronous	14	30
Number of liver metastases			
	1	28	61
	2	15	33
	3+	3	7
Recurrent disease			
	Any recurrence	29	63
	Hepatic recurrence	22	48
Liver metastasis histological differentiation			
	Well	1	2
	Moderate	40	87
	Poor	1	2
	NA	4	9
NACT			
	Yes	15	33
	No	31	67
Response NACT (n=15)			
	Partial Response	3	20
	Stable Disease	10	67
	Progressive Disease	2	13
CRS parameters (n=38)			
	N+ primary CRC	20	53
	DFS CRC < 12 months	29	76
	Number CLM > 1	15	40
	Size CLM > 5 cm	4	11
	CEA > 200	1	3
CRS (n=38)			
	0-2	29	76
	3-4	9	24

n=46 unless stated otherwise. Numbers represent frequency and percentages, except for age given in median and range.

ECOG, Eastern Cooperative Oncology Group; T-stage, primary tumor stage; N-stage, primary tumor lymph node status; CRC, colorectal cancer; CLM, colorectal liver metastases; CRS, clinical risk score; DFS, disease free survival; NACT, neoadjuvant chemotherapy.

### Mutations detected by targeted deep sequencing

**Figure 1:**
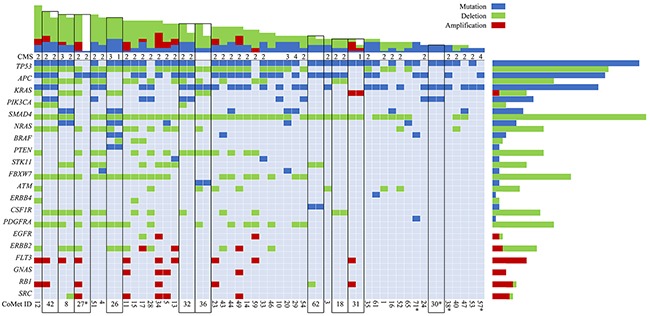
Somatic mutations and copy number alterations (CNAs) in colorectal liver metastases Main panel: Selected genes (y-axis) with mutations (blue), amplifications (red) and deletions (green); Oslo-CoMet trial sample ID (x-axis). Black outlines indicate metastatic pairs. Consensus molecular subtype (CMS) is indicated when available. Top panel: Total number of mutations, amplifications and deletions per sample. Right panel: Total number of mutations, amplifications and deletions for selected genes. *, CNA data not available.

Somatic mutations in the screened 50 cancer-related genes were detected in all but one patient (in 55 of 56 metastases). The most commonly mutated gene was *TP53*, detected in 35/46 (76%) of patients, followed by *APC* in 28/46 (61%) and *KRAS* in 27/46 (59%) cases (Figure 1A). In addition, mutations were detected in *PIK3CA 9/46* (20%), *SMAD4* 7/46 (15%), *NRAS* 5/46 (11%) and *BRAF* 3/46 (7%). Interestingly, the activating *BRAF* mutation p.V600E was not observed in any of the samples. The spectrum of driver mutations displayed large overlap compared with mutations detected in primary tumors (The Cancer Genome Atlas database) [10].

### Copy number alterations

**Figure 2:**
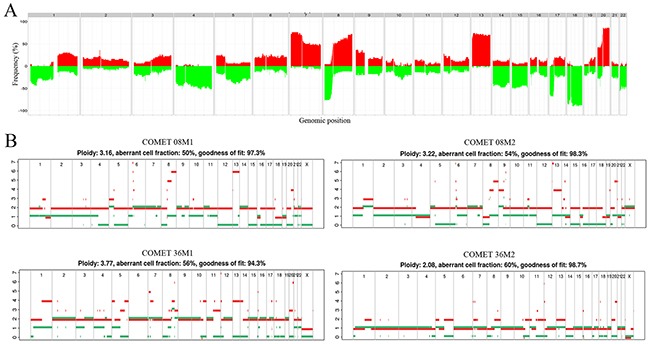
Genome wide copy number alterations **(A)** Frequency plot of genome wide copy number alterations. The histogram shows percentage of samples with specific alterations. The genomic position is indicated by chromosome 1 on the left and up to chromosome 22 on the right. Copy number gains for each region are depicted in red, and copy number losses are depicted in green. The plot shows high frequency of CNAs in chromosomes 7, 8, 13, 18 and 20. **(B)** Metastatic pairs displaying discrepancies in copy number profiles. Estimated ploidy and purity (tumor cell fraction) values are listed at the top of the plot. Metastatic pair 8M1 and 8M2 show discrepancies in segments of chromosomes 4, 13 and 14; metastatic pair 36M1 and 36M2 differ in total ploidy, with 36M1 having ploidy close to 4n and 36M2 having ploidy close to 2n.

In CLM, chromosomes 7, 13, and 18 were most frequently affected by copy number alterations (CNA), followed by chromosomes 14, 17 and 20, which is comparable to findings in primary CRC [11–13] (Figure 2A). Chromosome 18 had a frequently deleted region on the q-arm containing among others the *SMAD* gene family. Individual gene deletions and amplifications are presented in Figure 1. A large number of gene deletions were detected: *SMAD4* in 37/42 (88%), *TP53* 29/42 (69%), *FBXW7* 20/42 (48%), *PDGFRA* 16/42 (38%), *APC* 15/42 (36%), *NRAS* 14/42 (33%), *PTEN* 13/42 (31%), *CSF1R* 11/42 (26%), *ATM* 8/42 (19%) and *STK11* in 8/42 (19%) of patients. *FLT3* amplification was seen in 10/42 (24%) of patients. The following genes were found to be amplified or deleted: *ERBB2* amplification 3/42 (7%), deletion 8/42 (19%); *SRC* amplification 5/42 (12%), deletion 1/42 (2%); *KRAS* amplification 1/42 (2%), deletion 5/42 (12%); and *EGFR* amplification 2/42 (5%), deletion 1/42 (2%).

### Mutations and CNAs in metastatic pairs

DNA from two CLM removed during the same surgical procedure was available for analysis from ten patients. The initial mutation analysis revealed a discrepancy for only one metastatic pair (CoMet ID 30; Figure 1, Supplementary Table 2), showing a low read frequency (3%) of mutations in *TP53*, *KRAS* and *PIK3CA* in one metastasis and no somatic mutations in the other. On re-examination of the raw data, the same mutations were present with a read frequency below the predefined threshold of 2%. This specific patient had responded well to NACT with extensive fibrosis in the tumor samples used for analysis, suggesting that the discrepancy was caused by low tumor content. Thus, all pairs displayed identical mutations detectable by our panel. When investigating genome-wide CNA profiles, differences were detected in eight of the ten metastatic pairs (2 pairs, CoMet ID 27 and 30, were excluded from analysis due to suboptimal solution provided by the Allele-Specific Copy Number Analysis of Tumors (ASCAT) algorithm [14] probably as a result of low tumor content). For most of the pairs (n = 6), the differences were localized to particular segments, whereas for two samples, an overall shift in the ploidy was noticed (Figure 2B).

### Clustering-based transcriptome analyses of CLM and matched tumor-adjacent liver tissue

To stratify CLM based on gene expression, we first examined the relationship between the samples and the CMS classification recently defined by Guinney *et al* [1]. The distribution of subtypes assigned by the CMS classifier showed a high prevalence of the CMS2 subtype (30 out of 44 samples). The remaining samples were CMS1 (n=1), CMS4 (n=2), while 11 samples could not be assigned with sufficient certainty. The nearest centroid for 7 out of the 11 unclassified samples was CMS2. Hence, our cohort appeared to be highly enriched for CMS2 (37/44) and was relatively homogenous with respect to expression of the classifier genes defining primary CRC.

**Figure 3:**
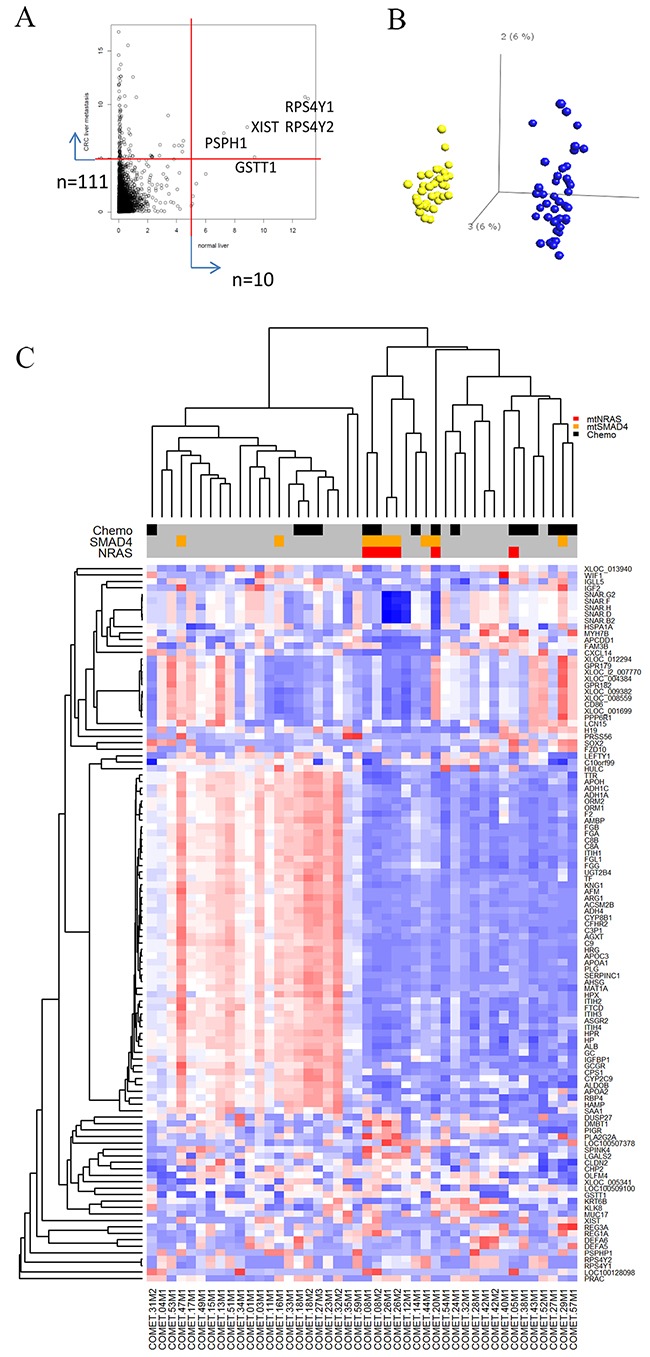
Gene expression variability in tumor-adjacent liver samples and metastases **(A)** Dot-plot showing distribution of variability of individual transcripts in tumor-adjacent liver samples (x-axis) and metastases (y-axis). Ten genes in tumor-adjacent liver samples and 111 genes in metastases were identified by a variance filter of the respective gene expression data sets (variance>5). **(B)** Principle component analysis comparing gene expression from tumor-adjacent liver samples (yellow dots) compared to metastases (blue dots). The two sample types formed distinct non-overlapping clusters. **(C)** Heat map representing the expression profiles of the 111 genes found to be highly variable in metastases and subjected to unsupervised clustering analysis. Red-blue scale: red represents high expression and blue low expression. Annotation of samples includes administration of neoadjuvant chemotherapy (NACT) and mutations in *SMAD4* and *NRAS*.

As an alternative approach to classify the samples, we performed hierarchical clustering based on genes with highly variable expression selected by variance filtering (var>5). The filter identified 10 highly variably expressed genes in tumor-adjacent liver samples, while 111 genes were highly variably expressed across the metastases (Figure 3A). In principal component analysis (PCA) plots, tumor-adjacent liver samples formed a highly distinct cluster when compared to the metastases, indicating profound differences in the transcriptomes of metastases and liver tissue (Figure 3B). Only five genes overlapped and reflected e.g. gender differences (*XIST, RPS4Y1, RPS4Y2*).

Hierarchical clustering of the metastases by the 111 most variable genes generated two distinct clusters displaying inverse expression pattern of a subset of 55 genes, designated 55^high^ and 55^low^ (Figure 3C and Supplementary Data set 1). Identification of significantly enriched canonical pathways associated with this focused set of 55 genes included “Acute Phase Response Signaling” (p-value=8.2E-37), “FXR/RXR Activation” (p-value=1.3E-33), “Coagulation System” (p-value=3.1E-12), and “IL-12 Signaling and Production in Macrophages” (p-value=4.9E-08) (Supplementary Data set 2). Functional annotation of these genes further delineated their role in cholesterol homeostasis (*APOA1, APOA2, CYP2C9, SAA1, PLG*) and blood coagulation (*APOH, F2, FGA, FGB, PLG, SERPINC1*). No differences in tumor cell content were found between the tumors in the two metastatic clusters by either histology or ASCAT estimation (Supplementary Figure 2A); thus, the clustering result could not be explained by differences in tumor/normal cell ratio. The 55^low^ sample cluster contained overrepresentation of *NRAS* mutated metastases (p=0.01) and metastases exposed to NACT (p=0.02). There were no differences in patient outcome when comparing these two groups. In order to validate the binary clustering pattern based on the 55 gene set, we clustered 2 publicly available datasets containing gene expression of CRC liver metastases (GSE14297 [15], GSE5851 [16]) which confirmed the division of CLM into 55^high^ and 55^low^ groups (Supplementary Figure 2B).

### Gene expression profiles associated with mutations and CNAs

The presence of mutations or CNAs in the most common oncogenic drivers (e.g. *APC, KRAS, BRAF, PIK3CA, FLT3, FBXW7*) was not associated with specific transcription signatures as no differentially expressed genes (DEGs) were identified by the R/BioConductor package, Linear Models for Microarray Data (LIMMA) analysis [17]. However, a set of 34 DEGs was associated with the presence of *TP53* “double-hit” (*TP53* mutation and *TP53* deletion (n=21; Supplementary Data set 3). Among these genes, down-regulated inhibitors of WNT-signaling (*APCDD1*, *FOXL1*), BMP-signaling (*SOSTDC1*), and up-regulated WNT target genes (*CHP2*, *CLDN2*) were identified. High levels of WNT-signaling and inverse role of BMP-signaling has been associated with loss and/or aberrant *TP53* expression in CRC [18].

For less frequent variants or aberrations in our cohort, 44 DEGs were identified when comparing cases with mutated (mt) *NRAS* and wild type (wt) *NRAS*, 22 DEGs were identified when comparing patients with or without mt*SMAD4*, and 59 DEGs were associated with *ERBB2* gene amplification (Supplementary Data set 4-7). The presence of activating mutations in *NRAS* was associated with up-regulation of 36 and down-regulation of 8 genes (Supplementary Data set 4). Up-regulated genes were downstream targets of interferon and pro-apoptotic activity, such as *IFIT1*, *IFI44L*, *CASP1*, *IFIT3*, *IRF9*. Interferon gamma (IFNG) and STAT1 were predicted as activated up-stream regulators of the 44 DEGs (Supplementary Data set 5).

Mutations in *SMAD4* were associated with increased expression of interferon inducible targets as with the mt*NRAS* profile (Supplementary Data set 6). However, DEGs also revealed up-regulated genes associated with worse prognosis and an aggressive phenotype, such as the poor prognosis marker *LY6E* [19] and chemotherapy resistance associated *UCP2* [20]. Down-regulated expression was observed for the tumor suppressor and inhibitor of WNT signaling *CXXC4*, and the differentiation inducing transcription factors, *HOXA7* and *HOXA9*.

### Gene expression profiles associated with clinicopathological parameters

Based on gender, 36 DEGs were identified, several overlapping with the gender-specific genes identified in the global variance analysis, as expected (Supplementary Data set 8). Interestingly, a number of genes encoded immune-related factors, which may be explained by the X chromosome containing a large number of immune-related genes. For example, the X chromosome contains 10% of all microRNA genes in the human genome, in contrast to none on the Y chromosome, and several of these microRNAs affect genes with immune-related function [21]. Right-sided CRC is associated with female gender, older age, higher tumor grade and poor prognosis. In our analysis, 50 DEGs were identified comparing CLM derived from right-sided (n=10) and left-sided (n=28) primary tumors (Supplementary Data set 9). Functional annotation of these genes revealed involvement of migratory and immune-related processes, with TGFB and TNF as upstream activated regulators (Supplementary Data set 10).

No significant differences were detected when comparing gene expression profiles of tumor-adjacent liver samples from patients who received NACT to patients who did not. In contrast, LIMMA analysis identified 208 DEGs between NACT treated (n=15) and non-treated (n=29) metastases (Supplementary Data set 11). The altered genes mapped to canonical pathways such as “Natural Killer Cell Signaling” (p-value=7.1E-06) and “Trem1 Signaling” (p-value=6.3E-05), “Dendritic Cell Maturation” (p-value=2.0E-04), and “Role of Pattern Recognition Receptors in Recognition of Bacteria/Viruses” (p-value=9.7E-04) (Supplementary Data set 12). These pathways are known to be involved in immunogenic cell death (ICD). In light of ICD, the data points to danger signaling through pattern recognition receptors (*e.g.* Toll-like receptors) amplified by TREM1-signaling, triggering an interferon directed response eliciting maturation of dendritic cells and natural killer cells. “Leukocyte migration” (p-value=2.7E-13) and related functions were predicted to be activated in NACT-exposed tumors [22, 23]. Furthermore, IFNG was assigned as the top activated upstream transcriptional regulator. IFNG typically mediates a pro-inflammatory response via STAT1-mediated induction of immune effectors such as *CD48*, *LCP1*, and *FCG3RA* observed in the DEG list. However, the predicted activation of STAT3 rather than STAT1 in the upstream analysis using Ingenuity Pathway Analysis (IPA), suggests a possible negative regulation of IFNG/STAT1 signaling, counter-balancing the inflammatory anti-tumor response. Interleukin 10, which is known to limit the extent of immune activation, also appeared as an upstream regulator. This provides a rationale for the observed identification of immune regulatory DEGs in the NACT-exposed metastases such as *CSF1R*, *CSF2,* *LAIR1*, *LILBR2,* *PIGR* and *HAVCR2.* Furthermore, the increased expression of *CD163* reflects macrophage polarization towards the anti-inflammatory M2-like macrophage phenotype.

### Associations between clinicopathological and molecular parameters and survival

**Figure 4:**
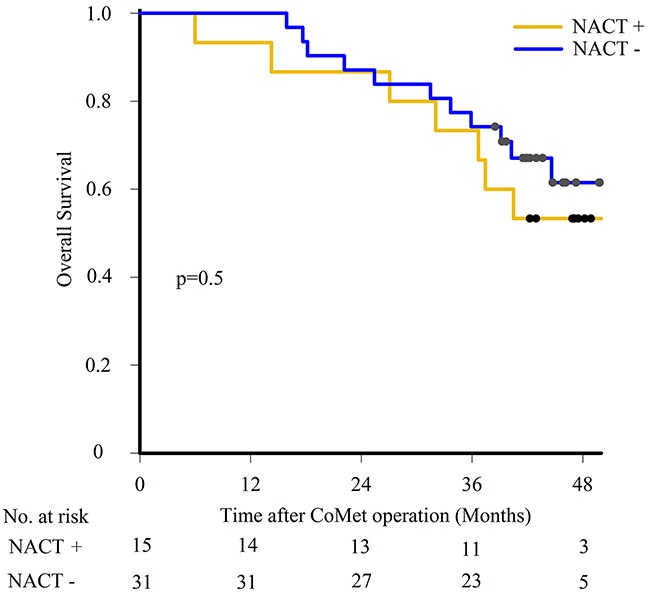
Survival outcome after liver metastasis surgery Kaplan-Meier estimates comparing overall survival using log-rank test in patients having neoadjuvant chemotherapy (NACT+) or not (NACT-).

Results from univariable analyses are shown in Table 2 and Supplementary Table 3, with selected survival curves in Figure 4. Survival analysis identified ECOG status hazard ratio (HR) 3.2, 95% confidence interval (CI) 1.3 - 8.2 for overall survival (OS) and HR 2.7, 95% CI 1.2 - 6.0 for disease free survival (DFS); and recurrence HR 6.0, 95% CI 1.4 - 26.1 for OS as significantly associated with outcome. In addition, females had a shorter DFS than males (17 months compared to 29 months; HR 2.4, 95% CI 1.2 - 5.1), while there was no gender difference in OS. No differences in OS and DFS were found comparing patients that did and did not receive NACT (Figure 4). From the molecular analyses, mt*SMAD4* was associated with shorter OS compared to wt*SMAD4* (HR 3.3, 95% CI 1.2 - 9.5), while the presence of *TP53, KRAS, APC* and *PIK3CA* mutations were not associated with differences in outcome.

**Table 2:** Univariable Cox proportional hazard analysis of OS and DFS including number of DEGs

OS								DFS		
Variable(n)		Months (95% CI)	HR	95% CI	p-Value	Months (95% CI)	HR	95% CI	p-Value	DEG
Gender										36
	Male (27)	45 (41-48)	Ref			29 (23-36)	Ref			
	Female (19)	39 (31-45)	1.7	0.7 - 4.3	0.3	17 (9-26)	2.4	1.2 – 5.1	0.02	
ECOG										0
	0 (34)	45 (42-49)	Ref			28 (22-35)	Ref	22-35		
	1-2 (12)	33 (25-42)	3.2	1.3 – 8.2	0.01	13 (5-21)	2.7	1.2 – 6.0	0.01	
Recurrence										0
	No (17)	49 (46-52)	Ref			NA				
	Yes (29)	38 (33-43)	6.0	1.4 – 26.1	0.02	NA	NA			
CRS										0
	0-2 (29)	43 (39-47)	ref				Ref			
	3-4 (9)	33 (23-43)	1.8	0.6 – 5.2	0.3		1.4	0.6 – 3.4	0.4	
NACT										208
	No (31)	43 (39-47)	Ref			25 (19-32)	Ref			
	Yes (15)	40 (33-47)	1.4	0.5 – 3.6	0.5	22 (12-33)	1.2	0.6 – 2.6	0.6	
*TP53* double hit										34
	wt (22)	42 (37-47)	Ref			22 (14-30)	Ref			
	mt + del (24)	42 (37-47)	0.9	0.4 – 2.3	0.9	26 (19-34)	1.2	0.6 – 2.4	0.7	
*SMAD4*										22
	wt (39)	44 (40-47)	Ref			26 (20-32)	Ref			
	mt (7)	31 (21-42)	3.3	1.2 – 9.5	0.02	17 (3-30)	1.9	0.8 – 4.7	0.2	
*NRAS*										44
	wt (41)	43 (39-46)	Ref			24 (18-29)	Ref			
	mt (5)	36 (20-52)	1.3	0.3 – 5.8	0.7	30 (13-47)	0.8	0.2 – 2.6	0.7	

Values are given in means.

OS, overall survival; DFS, disease free survival; HR, hazard ratio; CI, confidence interval; ECOG, Eastern Cooperative Oncology Group; NACT, neoadjuvant chemotherapy; mt, mutated; wt, wild type; del, deletion of gene; DEG, differentially expressed genes; NA, not applicable. OS and DFS (months) are calculated by the Kaplan-Meier method, HR and p-values are derived from Cox proportional hazard regression analysis.

## DISCUSSION

Using targeted deep sequencing, oncogenic mutations were identified in all except one tumor in this CLM cohort, and mutations and CNA profiles were found to be similar to profiles reported for primary CRC. Analyzing CLM metastatic pairs originating from the same primary tumor revealed similar mutations but differences in CNA profiles. The absence of differences on the mutational level can be explained by the focused gene panel used for targeted sequencing, and a broader panel might have revealed other discrepancies. The detected CNA differences show that individual metastases harbor characteristics that may be a result of cancer evolution which could contribute to the common clinical observation that individual metastatic tumors in the same patient may respond differently to treatment. The relatively small cohort size and inclusion of resectable CLM only might limit the generalizability of our results. In addition, for some of the features observed, the low number of events limits their power and requires larger cohorts to refine associated signatures and validate.

When applying the CMS classifier to gene expression data, the vast majority of samples were assigned to the “canonical” CMS2 subtype [1]. The clinical and molecular parameters in the CoMet samples, including gender, CRC anatomical location, and mutation frequency of *TP53* and *APC* are in accordance with the CMS2 profile, while *KRAS* mutations were more frequent in the CoMet cohort compared what is typically found in CMS2. Furthermore, CMS2 tumors have been characterized by WNT-signaling activation which is consistent with involvement of *TP53* mutations/aberrations and activation of WNT-signaling apparent in the gene expression results. The observed enrichment of CMS2 may reflect that the cohort was composed of patients with resectable CLM, good performance status, low CRS, and stable disease or partial response to NACT, all associated with a favorable prognosis. Importantly, the homogenous CMS classification result obtained suggests that the molecular features of this classification tool may be less useful when studying resectable CLM than primary CRC.

In the pursuit to define alternative subgroups in the CLM cohort, we identified a set of 55 genes that segregated the samples into two main subgroups. The segregation pattern was replicated in two publicly available CLM data sets, but the biological significance of the signature is not evident. The signature genes were associated with cholesterol homeostasis and acute inflammatory response, which strongly suggests that elements of the tumor-host microenvironment, particularly hepatocytes and infiltrating immune cells, are likely contributors. Intriguingly, the emerging role of lipid metabolism as a source of communication between cancer cells and infiltrating immune cells may be reflected in the signature genes. Among the top canonical pathways, we found the liver X receptor (LXR)/retinoid X receptor (RXR) and farnesoid X receptor (FXR)/RXR pathways. There is accumulating evidence that these nuclear receptor pathways play a tumor suppressive role in cancer [24] activating target genes that not only regulate lipid homoeostasis but also modulate inflammatory responses [24, 25]. The identified high-density lipoprotein component *APOA1,* is an LXR/RXR target known to control cholesterol efflux but which also has immune modulating activities, such as promoting polarization of tumor-associated macrophages towards the anti-tumor M1-like macrophage [26]. Similarly, the identified FXR/RXR pathway target *HRG* also promotes tumor-associated macrophage polarization towards the M1-like macrophage phenotype. Hence, this CLM cohort appears to be characterized by two distinct phenotypes with either apparent normal (55^high^) or dysregulated (55^low^) cholesterol/lipid homeostasis with reciprocal links to distinct immune profiles. These findings support the important role of metabolic alterations in shaping the immune microenvironment. The 55-gene signature was not associated with clinical outcome, and the two validation cohorts did not provide outcome data, suggesting that further studies in a larger cohort would be needed to determine the clinical utility of the signature.

Interestingly, when comparing gene expression in metastases from patients that had and had not received NACT, DEGs related to pathways associated with ICD were identified. Supporting the presence of ICD, the signature associated with NACT exposure comprised genes related to danger signaling through toll-like receptors, IFN response, and recruitment and activation of tumor infiltrating leukocytes, including natural killer cells and dendritic cells. In ICD, emission of danger signals from dying tumor cells elicit immune responses by presentation of tumor-derived antigens, engaging both the innate and adaptive immune system. Notably, oxaliplatin, which is a drug with documented ICD effects [27] was a component of NACT in 11 of 15 cases in this cohort. The immune effects induced by chemotherapy have been revealed in a number of tumor models but there is to our knowledge only one prior report describing an ICD-like signature in post-chemotherapy metastatic samples [28]. The concept that chemotherapy can invoke anti-tumor activity through ICD has become particularly pertinent because of the potential synergy with immune-based therapy [29–31]. However, the transcriptomic “snapshot” of NACT-exposed and non-exposed metastases rendered a more complex image, as it also featured expression of genes with immune regulatory and suppressive functions with STAT3 as a predicted up-stream regulator, indicating activation of processes that limit anti-tumor immunity [32]. To interpret these contrasting findings, an important aspect to consider is the substantial time lag (median 8 weeks) between NACT exposure and surgery. It is possible that NACT-induced prolonged signaling of ICD associated genes from tumor cells could trigger balancing immune suppressive mechanisms. Supporting our observations are analyses of post-treatment metastatic biopsies from high-grade serous ovarian cancer patients treated with platinum-based chemotherapy, in which an enhanced host immune response was detected following NACT, but evidence was also found that the effect was tempered by the co-occurrence of increased levels of immune checkpoint molecules (PD-1, CTLA4 and PD-L1) [28].

Although immune-related gene signatures were highly enriched in these analyses, the clinical interpretation is not clear, and further understanding of the dynamic co-evolvement of tumor and immune cells is needed in order to effectively exploit the immune system in treatment of CLM. Involvement of the immune system is not a surprising finding in CRC, as immune-related genes play a major role in CRC carcinogenesis by mediating inflammation, immune surveillance and evasion, and the presence of infiltrating immune cells and immune-related gene signatures is associated with prognosis in both primary CRC and CLM [33–37]. Immune modulating treatment, particularly the check-point inhibitors have, with the exception of microsatellite instable tumors (CMS1 subgroup), not been successful in treatment of metastatic CRC [38, 39]. The immune response activation in CLM identified by this study seems to be balanced by feedback mechanisms and immune escape, for example following NACT. This suggests that therapy aimed to overcome the immunosuppressive circuitries could be pursued in combinatory regimens in order to relieve the negative feedback loop controlling excessive anti-tumor immune responses and engage the immune system effectively. As a consequence, this may permit a broader clinical utility of immune therapy in CLM by inclusion of subgroups that could benefit from immune therapies in combinations with ICD-promoting agents.

## MATERIALS AND METHODS

### Patients

Patients with CLM suitable for local resections of less than three consecutive liver segments were eligible for inclusion in the Oslo-CoMet trial after evaluation by a multidisciplinary team. Patients with resectable extrahepatic disease were included, while patients who required formal hemihepatectomies were excluded [8]. One or two metastatic tumor samples and tumor-adjacent liver tissue from the first 71 patients included between February 2012 and April 2013 were available for molecular analyses. From these, a total of sixteen patients were excluded from analyses for the following reasons: unresectable tumors (n=2), benign lesions (n=4; 2 hemangiomas, 1 focal nodular hyperplasia and 1 fatty infiltration), missed lesions at surgery (n=2), no tissue for biobanking (n=7), not analyzed (n=1). Of the 55 patients submitted for molecular profiling, 9 had inadequate tumor content (<10%) when assessed microscopically, leaving materials for analyses from 46 patients (56 metastases, including 10 patients with metastatic pairs, and 46 tumor-adjacent liver samples) which constitute the study population. Targeted sequencing was successfully performed in 46 patients (56 individual metastases), CNA analyses in 42 patients (50 metastases, 6 metastases excluded because of low tumor content), and gene expression analyses in 38 patients (44 metastases, 12 metastases excluded because of poor RNA quality) and 37 tumor-adjacent liver samples.

### NACT

Fifteen of the 46 patients (33%) received NACT: 14 patients received fluoropyrimidine based therapy (10 in combination with oxaliplatin and 2 with irinotecan), and one patient had oxaliplatin monotherapy. No patient received anti-epidermal growth factor receptor treatment prior to surgery. The median number of chemotherapy cycles was 3 (1-10 cycles) over a median of 7 weeks (3-24 weeks), with a median of 8 weeks (3–19 weeks) from the last chemotherapy dose to liver resection. The effect of NACT was scored retrospectively on contrast enhanced computer tomography (CT) images by the study radiologist (AEB) according to the Response Evaluation in Solid Tumor 1.1 (RECIST 1.1) criteria using baseline and the pre-operative evaluation CT scans.

The study was approved by the Regional Committee for Health and Research Ethics in Norway (2011/1285/REK Sør-Øst B); by the Data Protection Official for Research at Oslo University Hospital in Norway, and written informed consent was required for participation, including consent for publishing data. Patient data was prospectively registered in the study database. Follow-up was scheduled every 4 months for the first 2 years and biannually from the next 3 years at Oslo University Hospital or at the referring hospital, and the censoring date for survival analyses was 15^th^ of May 2016. Date of death was obtained from the Norwegian National Registry. CRS [9] was calculated for patients having the CoMet operation as their first hepatic resection for CLM (n=38), assigning 1 point for each of the following parameters: lymph node positive primary CRC, disease free interval <12 months between primary CRC surgery and diagnosis of CLM, Carcinoembryonic antigen >200 μg/L at the time of liver resection, >1 liver metastases, largest metastasis >5 cm; giving a maximum score of 5.

### Tissue processing

Tumor and tumor-adjacent liver tissue samples were snap frozen in liquid nitrogen immediately after resection and stored at -80°C. Two frozen sections per tumor sample were assessed for tumor content by the study pathologist (KG), Samples with tumor content 10-100% (median 63%) were homogenized and aliquoted for further analysis. For tumor-adjacent liver tissue samples, the presence of liver tissue only was confirmed prior to processing.

### DNA isolation and next-generation targeted sequencing

DNA was isolated by AllPrep DNA/RNA MiniKit (Qiagen) using the QiaCube system according to the manufacturer's instructions. DNA was quantified by a NanoDrop ND-1000 (Thermo Scientific) spectrophotometer. Next generation sequencing was conducted using the Ion AmpliSeq Cancer Hotspot Panel (v2) for targeted amplification of 207 amplicons covering ~2800 hotspot mutations in 50 cancer-related genes, Ion AmpliSeq Library Kit 2.0 for library preparation, Ion PGM OT2 200 Template Kit v2 DL and Ion OneTouch ES Instrument for emulsion PCR and enrichment, Ion PGM 200 Sequencing Kit v2, Ion 318 Chips, and the PGM sequencing platform (Life Technologies), as recommended by the manufacturers’ protocols without modification. The DNA input for amplicon library generation was 10 ng. Ten samples were barcoded using Ion Xpress Barcode Adapters (Life Technologies), pooled, and run on a single Ion 318 chip. Initial data from the PGM runs were processed by The Torrent Suite Variant Caller using panel customized parameters as provided by Life Technologies. Successful sequencing of a sample required at least 300 000 AQ20 reads. A minimum coverage of 500× with at least 2% frequency was used as cut-off for a variant to be considered true. Additional manual evaluation was used to exclude false positive variant calls.

### Analysis of CNAs

Somatic CNAs were analyzed using Genome-Wide Human SNP array 6.0 (Affymetrix). Raw data was normalized to HapMap by Affymetrix Power tools. Copy number profiles were obtained using ASCAT algorithm [14]. Following segmentation, the core ASCAT algorithm determines the fraction of non-aberrant cells and the tumor ploidy (the average number of DNA copies), and generates an ASCAT profile. Copy number profiles were successfully obtained for 50 of 56 metastases with sufficient tumor percentage. Subsequently, ASCAT results were used to obtain average CNA profiles for all tumor samples included in the study cohort, and for quantitative assignment of deletion or amplification of selected cancer-related genes from the AmpliSeq panel.

### Microarray analysis

Total RNA from fresh-frozen samples was isolated using TRIzol reagent (Invitrogen). The RNA quantity was determined using the NanoDrop ND-1000 spectrometer (Thermo Scientific), and RNA integrity numbers (RIN) were measured with an RNA 6000 Kit on the 2100 Bioanalyzer (Agilent Technologies) according to the manufacturer's protocol. Microarrays from Agilent Technologies (Agilent SurePrint G3 Human Gene Expression 8×60K v2) were used for mRNA profiling. Total RNA (100μg) was labelled with Cy3 and hybridized on the arrays according to the manufacturer's recommendations. Arrays were scanned using Agilent Microarray Scanner (Agilent Technologies). The raw signal data were pre-processed with Agilent's Feature Extraction Software (v10.7.3.1).

### RT-qPCR validation

Thirteen genes were selected for validation (Supplementary Table 1) by RT-qPCR. For cDNA synthesis, total RNA was reverse transcribed by SuperScript III First Strand Synthesis SuperMix (Invitrogen). Real time PCR reactions were conducted using *Power* SYBR® Green PCR Master Mix reagents (Applied Biosystems®) and Roche Light Cycler 480 system. Data was analyzed using *GAPDH* as the endogenous control gene. The results confirmed the identified differentially expressed genes (Supplementary Figure 1).

### Statistical and bioinformatics analysis

Variables were described using frequencies, percentages, mean with 95% CI. A binary variable for age was created, using the cohort´s median age as cut off. Univariable analyses were performed by the Kaplan-Meier method to estimate OS, defined from the time of CoMet surgery to death or the censoring date (15^th^ of May 2016), and DFS from CoMet surgery to the time of local recurrence, distant metastases or last follow-up. HR were derived using Cox proportional hazard analysis, and 95% CI and p-values are reported. Multivariable analyses were not performed. A p-value <0.05 was considered statistically significant. The SPSS software (version 21.0, IBM SPSS, Chicago, IL, USA) was used for calculations.

Gene expression data was log_2_-transformed and quantile normalized using the LIMMA [17]. Imputation of missing values was performed by local least squares (llsImpute from the R package pcaMethods [40]) with k=20. For subgroup discovery and visualization, data were assessed using a two-way, unsupervised average linkage hierarchical clustering on genes showing high overall variability (var>5.0) using the R packages ctc [41] and heatmap.plus [42]. LIMMA was used on variance filtered data (var>0.5) to identify DEGs and the false discovery rate-adjusted p-value ≤0.1 was used as the cut-off for selecting significant genes. In cases where two individual metastatic samples were available, one randomly selected sample per patient was applied in LIMMA analysis with analysis of NACT as an exception. Here we regarded the metastatic pairs as independent samples based on the extrinsic nature of NACT exposure. Differentially expressed genes were imported into IPA software (Ingenuity Systems) for pathway and functional analysis. Significance of each pathway and functional group was assessed by IPA using the Fisher's exact tests (p-value ≤0.05). Upstream transcriptional regulation was predicted by IPA through the Activation z-score statistic. The predicted regulatory relationships are associated with a direction of change that is either activating (z-score ≥2) or inhibiting (z-score ≤-2). CMS was assigned using the “CMSclassifier” R package [1] which is based on a similarity-to-centroid approach using centroids of the CMS calculated from 693 discriminant genes.

## CONCLUSION

The results from this study underline that although CLM resemble primary CRC, understanding the molecular features of metastatic tumors may provide knowledge with biological and clinical relevance. Importantly, the CMS classification that has provided a valuable tool in primary CRC may not be equally useful for classification of CLM. Immune-related and metabolic gene expression signatures were identified by hierarchical clustering of the most variable genes and in tumors from patients receiving NACT, suggesting an opportunity for immune modulating strategies in subgroups of CLM. It seems of particular interest to explore immune therapy in combination with NACT regimens to exploit possible synergy with ICD.

## SUPPLEMENTARY MATERIALS FIGURES AND TABLES









## Abbreviations

ASCAT: Allele-specific copy number analysis of tumors
CI: Confidence interval
CLM: Colorectal liver metastases
CMS: Consensus molecular subtypes
CNA: Copy number alterations
CRC: Colorectal cancer
CRS: Clinical risk score
DEG: Differentially expressed genes
DFS: Disease free survival
ECOG: Eastern Cooperative Oncology Group
FDR: False discovery rate
HR: Hazard ratio
ICD: Immunogenic cell death
IFNG: Interferon gamma
IPA: Ingenuity pathway analysis
LIMMA: Linear model for microarray data
mt: Mutated
NACT: Neoadjuvant chemotherapy
OS: Overall survival
PCA: Principal component analysis
RECIST: Response evaluation criteria in solid tumors
wt: Wild type
