# Supplementary material for: Molecular signatures reflecting microenvironmental metabolism and chemotherapy-induced immunogenic cell death in colorectal liver metastases
Source: Oncotarget. 2017 Jul 18;8(44):76290–304. doi: 10.18632/oncotarget.19350 (PMC5652706; doi:10.18632/oncotarget.19350)
Supplement: Supplementary file 1 [file oncotarget-08-76290-s001.pdf]

Molecular signatures reflecting microenvironmental metabolism and chemotherapy-induced immunogenic cell death in colorectal liver metastases

SUPPLEMENTARY MATERIALS

Supplementary Table 1: Primers used for RT-qPCR validation

Gene symbol	ID (Pubmed)	Forward primer		Reverse primer	
AXIN2	**6653583**	**2066**	**AGTCAGCAGAGGGACAGGAA**	**2182**	**GTGGACACCTGCCAGTTTCT**
APC2	57242791	8769	ACCCCCAGTTAACACTGCTG	8888	GGCACACCTCGGTGACTTAT
DMBT1	157423469	4259	ATCTCCAATCCTTGGGCTTT	4380	GCAGCCTGAGTAGGGAATTG
PLA2G2A	13543520	765	CCCTCCCTACCCTAACCAAG	882	GCTGGAAATCTGCTGGATGT
TGFBR3	223459585	1445	ACCCTGTCATTCCCAGCATA	1561	CAGCCACGATCATCTTCTCA
HOXA7A	84105267	1216	TATCCCAACACTGGCAGACA	1339	GGCACTAGGTAGCAGGCAAG
CECR1	30704989	2572	TCCCATAGCTGGTGAAGGAC	2692	GATGAGGGATGCGTTCTGAT
CSF1R	28833090	966	CCAAAAAGTCCTGACCCTCA	1086	CTCTACCACCCGGAAGAACA
FABP6	894182	146	CGCAACTTCAAGATCGTCAC	264	ATGTTGCTTTCCTTGCCAAC
TFF3	17389673	349	CAGGCACTGTTCATCTCAGC	455	GAGCATGGGACCTTTATTCG
TRIM7	124376611	1937	AAGCCAGTCCTGCTGTTCTC	2059	GAGGTCCCACAACCTCTCAA
PLA2G2F	145553988	1234	GTGGCTACTTTGGGCTTGAA	1356	ACACTCAGCACTGCACATCC
GAPDH	53734501	1045	GGCCTCCAAGGAGTAAGACC	1166	AGGGGAGATTCAGTGTGGTG
CHKA	34192558	1187	GCCTGCATTCCAAAATGACT	1301	TCCCCAGAGGAAATGAGATG

Supplementary Table 2: Somatic variants

See Supplementary File 1

Supplementary Table 3: Univariable Cox proportional hazard analysis of OS and DFS, including DEGs

See Supplementary File 2

Supplementary Data_set1-12: Gene expression data

See Supplementary File 3

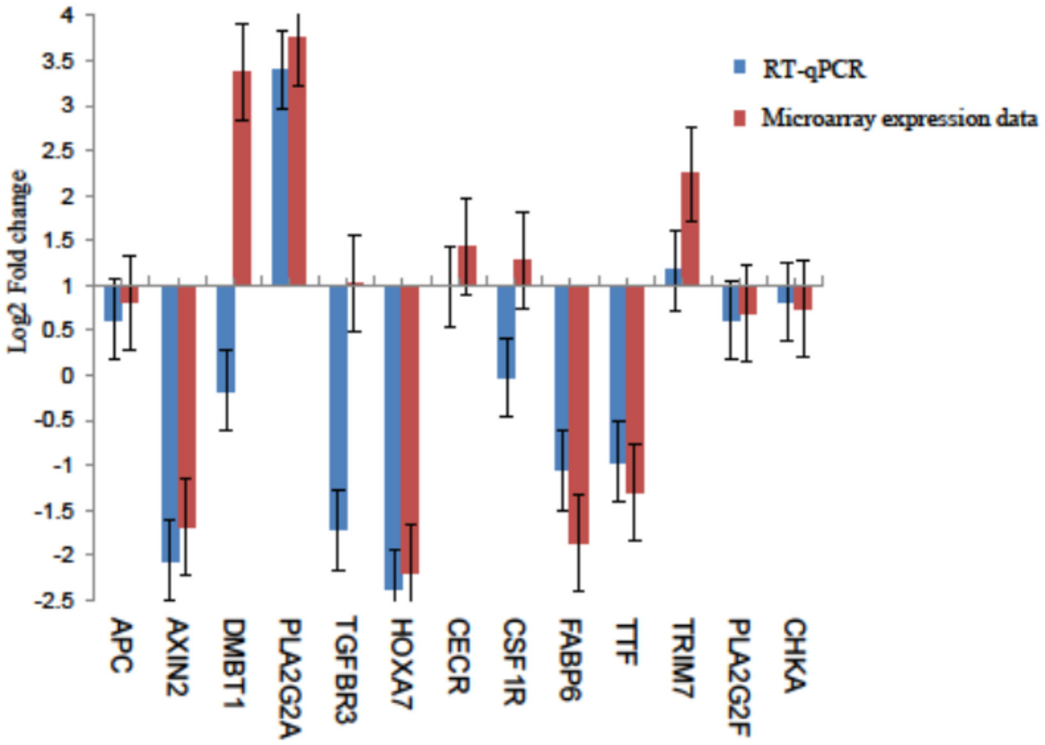

Supplementary Figure 1: RT-qPCR Validation. Validation of differentially expressed genes by RT-qPCR. Log_2 fold changes in microarray data (red bars) were validated by RT-qPCR (blue bars) using *GAPDH* as a reference gene.

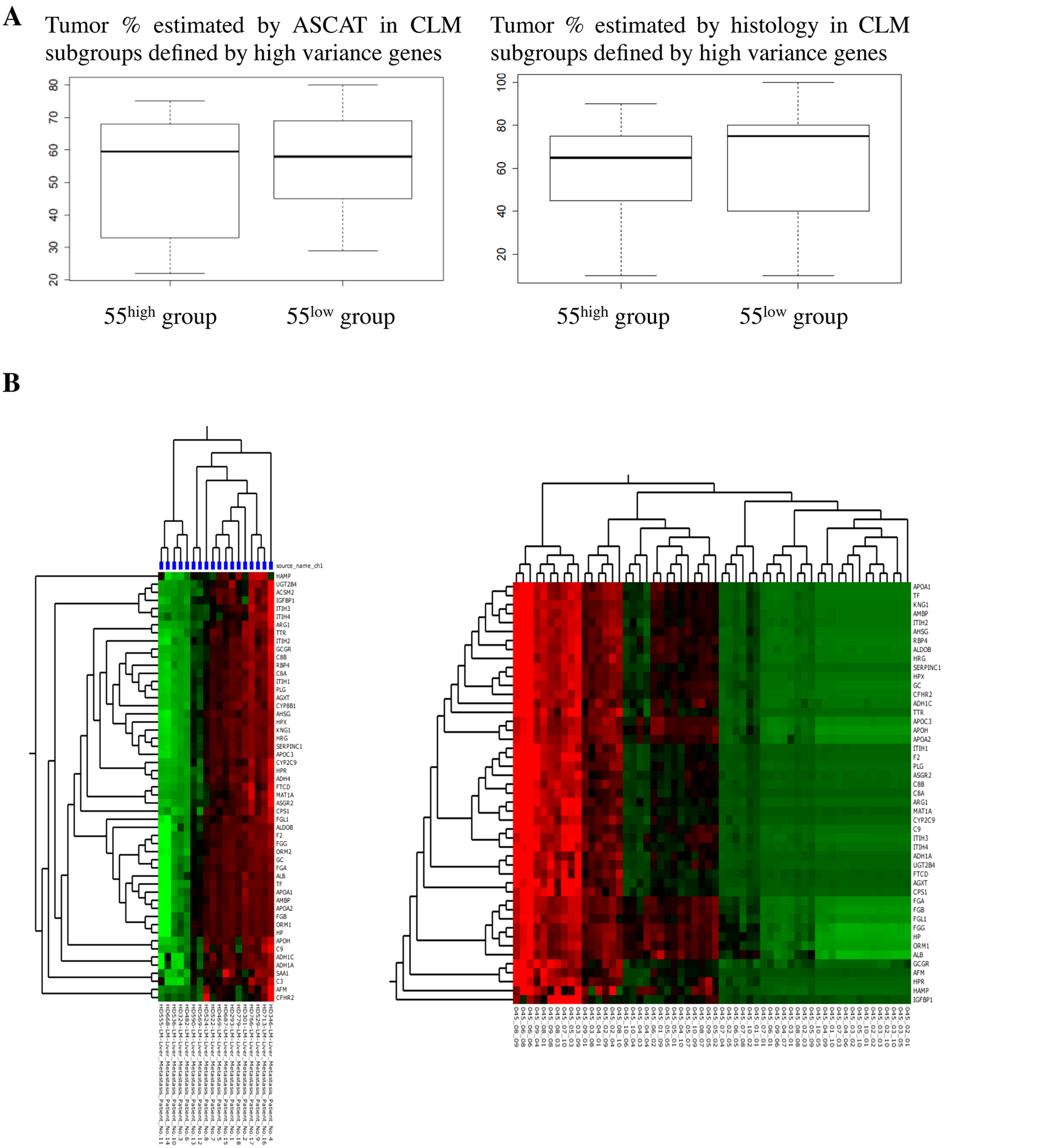

Supplementary Figure 2: Tumor percentage estimation. **(A)** Estimation of tumor percentage in samples by ASCAT and histology in the two subgroups of metastases differing by high or low expression of 55 genes exhibiting high variance in the gene expression data set. Neither estimate showed significant differences in tumor content between the two groups. **(B)** Expression pattern of the 55 gene cluster in CLM in two publicly available cohorts (GSE14297 [15], GSE5851 [16]). The samples/genes were subjected to clustering analysis. Red-green scale: red represents high expression and green low expression. The expression of the 55 genes is commonly up- or down-regulated, forming two main subgroups and parallel to the CoMet gene expression pattern.
