# Supplementary material for: Molecular signatures reflecting microenvironmental metabolism and chemotherapy-induced immunogenic cell death in colorectal liver metastases
Source: Oncotarget. 2017 Jul 18;8(44):76290–304. doi: 10.18632/oncotarget.19350 (PMC5652706; doi:10.18632/oncotarget.19350)
Supplement: Supplementary file 2 [file oncotarget-08-76290-s002.docx]

**Supplementary table 2:** **Somatic variants**

| **SampleID** | **TargetGene** | **Protein change** | **CDS change** | **Change** | **RefSeqRNA** | **Function** | **Functional change** |
| --- | --- | --- | --- | --- | --- | --- | --- |
| COMET-0001M1-1 | KRAS | p.G12V | c.G35T | Chr12:g.25398284:C>A | NM_033360.2 | exonic | nonsynonymous SNV |
| COMET-0003M1-1 | APC | p.R876X | c.C2626T | Chr5:g.112173917:C>T | NM_000038.4 | exonic | stopgain SNV |
|  | KRAS | p.Q61H | c.A183C | Chr12:g.25380275:T>G | NM_033360.2 | exonic | nonsynonymous SNV |
|  | TP53 | p.Q100X | c.C298T | Chr17:g.7579389:G>A | NM_000546.5 | exonic | stopgain SNV |
| COMET-0004M1-1 | FBXW7 | p.R505C | c.C1513T | Chr4:g.153247289:G>A | NM_033632.3 | exonic | nonsynonymous SNV |
|  | APC | p.P1440fs | c.4318_4319insCA | Chr5:g.112175609:->CA | NM_000038.4 | exonic | frameshift insertion |
|  | KRAS | p.G13D | c.G38A | Chr12:g.25398281:C>T | NM_033360.2 | exonic | nonsynonymous SNV |
| COMET-0005M1-1 | NRAS | p.G12D | c.G35A | Chr1:g.115258747:C>T | NM_002524.3 | exonic | nonsynonymous SNV |
|  | TP53 | p.R248Q | c.G743A | Chr17:g.7577538:C>T | NM_000546.5 | exonic | nonsynonymous SNV |
| COMET-0008M1-1 | NRAS | p.Q61K | c.C181A | Chr1:g.115256530:G>T | NM_002524.3 | exonic | nonsynonymous SNV |
|  | TP53 | p.C277X | c.T831A | Chr17:g.7577107:A>T | NM_000546.5 | exonic | stopgain SNV |
|  | SMAD4 | p.L495P | c.T1484C | Chr18:g.48604662:T>C | NM_005359.5 | exonic | nonsynonymous SNV |
| COMET-0008M2-1 | NRAS | p.Q61K | c.C181A | Chr1:g.115256530:G>T | NM_002524.3 | exonic | nonsynonymous SNV |
|  | TP53 | p.C277X | c.T831A | Chr17:g.7577107:A>T | NM_000546.5 | exonic | stopgain SNV |
|  | SMAD4 | p.L495P | c.T1484C | Chr18:g.48604662:T>C | NM_005359.5 | exonic | nonsynonymous SNV |
| COMET-0010M1-1 | PIK3CA | p.E545K | c.G1633A | Chr3:g.178936091:G>A | NM_006218.2 | exonic | nonsynonymous SNV |
|  | APC | p.E1577fs | c.4729delG | Chr5:g.112176020:G>- | NM_000038.4 | exonic | frameshift deletion |
|  | KRAS | p.G12S | c.G34A | Chr12:g.25398285:C>T | NM_033360.2 | exonic | nonsynonymous SNV |
|  | TP53 | p.R282W | c.C844T | Chr17:g.7577094:G>A | NM_000546.5 | exonic | nonsynonymous SNV |
| COMET-0011M1-1 | TP53 | p.L194R | c.T581G | Chr17:g.7578268:A>C | NM_000546.5 | exonic | nonsynonymous SNV |
| COMET-0012M1-1 | APC | p.I1307fs | c.3921_3925del | Chr5:g.112175212:AAAAG>- | NM_000038.4 | exonic | frameshift deletion |
|  | KRAS | p.G12V | c.G35T | Chr12:g.25398284:C>A | NM_033360.2 | exonic | nonsynonymous SNV |
|  | TP53 | p.R273H | c.G818A | Chr17:g.7577120:C>T | NM_000546.5 | exonic | nonsynonymous_SNV |
| COMET-0013M1-1 | APC | p.E1554fs | c.4660_4661insA | Chr5:g.112175951:->A | NM_000038.4 | exonic | frameshift insertion |
|  | TP53 | p.R196X | c.C586T | Chr17:g.7578263:G>A | NM_000546.5 | exonic | stopgain SNV |
|  | STK11 |  |  | Chr19:g.1220505:G>T | NM_000455.4 | splicing |  |
| COMET-0014M1-1 | APC | p.S1315X | c.C3944G | Chr5:g.112175235:C>G | NM_000038.4 | exonic | stopgain SNV |
|  | KRAS | p.G12D | c.G35A | Chr12:g.25398284:C>T | NM_033360.2 | exonic | nonsynonymous SNV |
|  | TP53 | p.L265P | c.T794C | Chr17:g.7577144:A>G | NM_000546.5 | exonic | nonsynonymous SNV |
| COMET-0015M1-1 | APC | p.E1317X | c.G3949T | Chr5:g.112175240:G>T | NM_000038.4 | exonic | stopgain SNV |
|  | TP53 | p.R213X | c.C637T | Chr17:g.7578212:G>A | NM_000546.5 | exonic | stopgain SNV |
| COMET-0016M1-1 | TP53 | p.R248Q | c.G743A | Chr17:g.7577538:C>T | NM_000546.5 | exonic | nonsynonymous SNV |
|  | TP53 | p.P72fs | c.215delC | Chr17:g.7579472:G>- | NM_000546.5 | exonic | frameshift deletion |
|  | SMAD4 | p.A532D | c.C1595A | Chr18:g.48604773:C>A | NM_005359.5 | exonic | nonsynonymous SNV |
| COMET-0017M1-1 | PIK3CA | p.E545K | c.G1633A | Chr3:g.178936091:G>A | NM_006218.2 | exonic | nonsynonymous SNV |
|  | APC | p.R1450X | c.C4348T | Chr5:g.112175639:C>T | NM_000038.4 | exonic | stopgain SNV |
|  | KRAS | p.G13D | c.G38A | Chr12:g.25398281:C>T | NM_033360.2 | exonic | nonsynonymous SNV |
|  | TP53 | p.H178fs | c.532_533insC | Chr17:g.7578397:->G | NM_000546.5 | exonic | frameshift insertion |
| COMET-0018M1-1 | APC | p.I1307fs | c.3921_3925del | Chr5:g.112175212:AAAAG>- | NM_000038.4 | exonic | frameshift deletion |
|  | TP53 | p.R273H | c.G818A | Chr17:g.7577120:C>T | NM_000546.5 | exonic | nonsynonymous_SNV |
| COMET-0018M2-1 | APC | p.I1307fs | c.3921_3925del | Chr5:g.112175212:AAAAG>- | NM_000038.4 | exonic | frameshift deletion |
|  | TP53 | p.R273H | c.G818A | Chr17:g.7577120:C>T | NM_000546.5 | exonic | nonsynonymous_SNV |
| COMET-0020M1-1 | NRAS | p.G12D | c.G35A | Chr1:g.115258747:C>T | NM_002524.3 | exonic | nonsynonymous SNV |
|  | FBXW7 | p.L589P | c.T1766C | Chr4:g.153245425:A>G | NM_033632.3 | exonic | nonsynonymous SNV |
|  | TP53 | p.R282W | c.C844T | Chr17:g.7577094:G>A | NM_000546.5 | exonic | nonsynonymous SNV |
|  | SMAD4 | p.F119L | c.T357A | Chr18:g.48575163:T>A | NM_005359.5 | exonic | nonsynonymous SNV |
| COMET-0023M1-1 | APC | p.I1307fs | c.3921_3925del | Chr5:g.112175212:AAAAG>- | NM_000038.4 | exonic | frameshift deletion |
|  | KRAS | p.G12V | c.G35T | Chr12:g.25398284:C>A | NM_033360.2 | exonic | nonsynonymous SNV |
|  | TP53 | p.R248W | c.C742T | Chr17:g.7577539:G>A | NM_000546.5 | exonic | nonsynonymous SNV |
| COMET-0024M1-1 | PIK3CA | p.Q546K | c.C1636A | Chr3:g.178936094:C>A | NM_006218.2 | exonic | nonsynonymous SNV |
|  | APC | p.G1428X | c.G4282T | Chr5:g.112175573:G>T | NM_000038.4 | exonic | stopgain SNV |
|  | KRAS | p.G12D | c.G35A | Chr12:g.25398284:C>T | NM_033360.2 | exonic | nonsynonymous SNV |
| COMET-0026M1-1 | NRAS | p.G12C | c.G34T | Chr1:g.115258748:C>A | NM_002524.3 | exonic | nonsynonymous SNV |
|  | BRAF | p.N581S | c.A1742G | Chr7:g.140453193:T>C | NM_004333.4 | exonic | nonsynonymous SNV |
|  | PTEN | p.R233X | c.C697T | Chr10:g.89717672:C>T | NM_000314.4 | exonic | stopgain SNV |
|  | TP53 | p.R306X | c.C916T | Chr17:g.7577022:G>A | NM_000546.5 | exonic | stopgain SNV |
|  | SMAD4 | p.R361C | c.C1081T | Chr18:g.48591918:C>T | NM_005359.5 | exonic | nonsynonymous SNV |
| COMET-0026M2-1 | NRAS | p.G12C | c.G34T | Chr1:g.115258748:C>A | NM_002524.3 | exonic | nonsynonymous SNV |
|  | BRAF | p.N581S | c.A1742G | Chr7:g.140453193:T>C | NM_004333.4 | exonic | nonsynonymous SNV |
|  | PTEN | p.R233X | c.C697T | Chr10:g.89717672:C>T | NM_000314.4 | exonic | stopgain SNV |
|  | TP53 | p.R306X | c.C916T | Chr17:g.7577022:G>A | NM_000546.5 | exonic | stopgain SNV |
|  | SMAD4 | p.R361C | c.C1081T | Chr18:g.48591918:C>T | NM_005359.5 | exonic | nonsynonymous SNV |
| COMET-0027M1-1 | APC | p.E1295fs | c.3884delA | Chr5:g.112175175:A>- | NM_000038.4 | exonic | frameshift deletion |
| COMET-0027M3-1 | APC | p.E1295fs | c.3884delA | Chr5:g.112175175:A>- | NM_000038.4 | exonic | frameshift deletion |
| COMET-0028M1-1 | PIK3CA | p.E545K | c.G1633A | Chr3:g.178936091:G>A | NM_006218.2 | exonic | nonsynonymous SNV |
|  | KRAS | p.G12V | c.G35T | Chr12:g.25398284:C>A | NM_033360.2 | exonic | nonsynonymous SNV |
| COMET-0029M1-1 | APC | p.Q1378X | c.C4132T | Chr5:g.112175423:C>T | NM_000038.4 | exonic | stopgain SNV |
|  | KRAS | p.G12C | c.G34T | Chr12:g.25398285:C>A | NM_033360.2 | exonic | nonsynonymous SNV |
|  | TP53 | p.G266E | c.G797A | Chr17:g.7577141:C>T | NM_000546.5 | exonic | nonsynonymous SNV |
|  | SMAD4 | p.P356T | c.C1066A | Chr18:g.48591903:C>A | NM_005359.5 | exonic | nonsynonymous SNV |
| COMET-0030M1-3 | PIK3CA | p.E542K | c.G1624A | Chr3:g.178936082:G>A | NM_006218.2 | exonic | nonsynonymous SNV |
|  | KRAS | p.G13D | c.G38A | Chr12:g.25398281:C>T | NM_033360.2 | exonic | nonsynonymous SNV |
|  | TP53 | p.R273H | c.G818A | Chr17:g.7577120:C>T | NM_000546.5 | exonic | nonsynonymous_SNV |
| COMET-0030M2-2* | PIK3CA | p.E542K | c.G1624A | Chr3:g.178936082:G>A | NM_006218.2 | exonic | nonsynonymous SNV |
|  | KRAS | p.G13D | c.G38A | Chr12:g.25398281:C>T | NM_033360.2 | exonic | nonsynonymous SNV |
|  | TP53 | p.R273H | c.G818A | Chr17:g.7577120:C>T | NM_000546.5 | exonic | nonsynonymous_SNV |
| COMET-0031M1-1 | TP53 | p.R273H | c.G818A | Chr17:g.7577120:C>T | NM_000546.5 | exonic | nonsynonymous_SNV |
| COMET-0031M2-1 | TP53 | p.R273H | c.G818A | Chr17:g.7577120:C>T | NM_000546.5 | exonic | nonsynonymous_SNV |
| COMET-0032M1-1 | PIK3CA | p.Q546K | c.C1636A | Chr3:g.178936094:C>A | NM_006218.2 | exonic | nonsynonymous SNV |
|  | APC | p.I1307fs | c.3921_3925del | Chr5:g.112175212:AAAAG>- | NM_000038.4 | exonic | frameshift deletion |
|  | KRAS | p.G12V | c.G35T | Chr12:g.25398284:C>A | NM_033360.2 | exonic | nonsynonymous SNV |
|  | TP53 | p.R175H | c.G524A | Chr17:g.7578406:C>T | NM_000546.5 | exonic | nonsynonymous SNV |
| COMET-0032M2-1 | PIK3CA | p.Q546K | c.C1636A | Chr3:g.178936094:C>A | NM_006218.2 | exonic | nonsynonymous SNV |
|  | APC | p.I1307fs | c.3921_3925del | Chr5:g.112175212:AAAAG>- | NM_000038.4 | exonic | frameshift deletion |
|  | KRAS | p.G12V | c.G35T | Chr12:g.25398284:C>A | NM_033360.2 | exonic | nonsynonymous SNV |
|  | TP53 | p.R175H | c.G524A | Chr17:g.7578406:C>T | NM_000546.5 | exonic | nonsynonymous SNV |
| COMET-0033M1-1 | APC | p.Q1444X | c.C4330T | Chr5:g.112175621:C>T | NM_000038.4 | exonic | stopgain SNV |
|  | KRAS | p.G13D | c.G38A | Chr12:g.25398281:C>T | NM_033360.2 | exonic | nonsynonymous SNV |
|  | TP53 | p.G245S | c.G733A | Chr17:g.7577548:C>T | NM_000546.5 | exonic | nonsynonymous SNV |
| COMET-0034M1-1 | APC | p.I1307fs | c.3921_3925del | Chr5:g.112175212:AAAAG>- | NM_000038.4 | exonic | frameshift deletion |
|  | TP53 | p.R273C | c.C817T | Chr17:g.7577121:G>A | NM_000546.5 | exonic | nonsynonymous SNV |
| COMET-0035M1-1 | APC | p.R1450X | c.C4348T | Chr5:g.112175639:C>T | NM_000038.4 | exonic | stopgain SNV |
|  | KRAS | p.G12D | c.G35A | Chr12:g.25398284:C>T | NM_033360.2 | exonic | nonsynonymous SNV |
|  | TP53 | p.R248Q | c.G743A | Chr17:g.7577538:C>T | NM_000546.5 | exonic | nonsynonymous SNV |
| COMET-0036M1-1 | ATM | p.R3008H | c.G9023A | Chr11:g.108236087:G>A | NM_000051.3 | exonic | nonsynonymous SNV |
|  | KRAS | p.G13D | c.G38A | Chr12:g.25398281:C>T | NM_033360.2 | exonic | nonsynonymous SNV |
| COMET-0036M2-1 | ATM | p.R3008H | c.G9023A | Chr11:g.108236087:G>A | NM_000051.3 | exonic | nonsynonymous SNV |
|  | KRAS | p.G13D | c.G38A | Chr12:g.25398281:C>T | NM_033360.2 | exonic | nonsynonymous SNV |
| COMET-0038M1-1 | APC | p.I1304fs | c.3911delT | Chr5:g.112175202:T>- | NM_000038.4 | exonic | frameshift deletion |
|  | KRAS | p.G12D | c.G35A | Chr12:g.25398284:C>T | NM_033360.2 | exonic | nonsynonymous SNV |
|  | TP53 | p.R196X | c.C586T | Chr17:g.7578263:G>A | NM_000546.5 | exonic | stopgain SNV |
| COMET-0040M1-1 | TP53 | p.M246K | c.T737A | Chr17:g.7577544:A>T | NM_000546.5 | exonic | nonsynonymous SNV |
| COMET-0042M1-1 | PIK3CA | p.E545K | c.G1633A | Chr3:g.178936091:G>A | NM_006218.2 | exonic | nonsynonymous SNV |
|  | APC | p.S1100fs | c.3298_3299del | Chr5:g.112174589:TC>- | NM_000038.4 | exonic | frameshift deletion |
|  | APC | p.Q1447X | c.C4339T | Chr5:g.112175630:C>T | NM_000038.4 | exonic | stopgain SNV |
|  | KRAS | p.G12S | c.G34A | Chr12:g.25398285:C>T | NM_033360.2 | exonic | nonsynonymous SNV |
|  | TP53 | p.P151T | c.C451A | Chr17:g.7578479:G>T | NM_000546.5 | exonic | nonsynonymous SNV |
| COMET-0042M2-1 | PIK3CA | p.E545K | c.G1633A | Chr3:g.178936091:G>A | NM_006218.2 | exonic | nonsynonymous SNV |
|  | APC | p.S1100fs | c.3298_3299del | Chr5:g.112174589:TC>- | NM_000038.4 | exonic | frameshift deletion |
|  | APC | p.Q1447X | c.C4339T | Chr5:g.112175630:C>T | NM_000038.4 | exonic | stopgain SNV |
|  | KRAS | p.G12S | c.G34A | Chr12:g.25398285:C>T | NM_033360.2 | exonic | nonsynonymous SNV |
|  | TP53 | p.P151T | c.C451A | Chr17:g.7578479:G>T | NM_000546.5 | exonic | nonsynonymous SNV |
| COMET-0043M1-1 | APC | p.S1495fs | c.4485delT | Chr5:g.112175776:T>- | NM_000038.4 | exonic | frameshift deletion |
|  | BRAF | p.G469V | c.G1406T | Chr7:g.140481402:C>A | NM_004333.4 | exonic | nonsynonymous SNV |
|  | TP53 | p.Q165X | c.C493T | Chr17:g.7578437:G>A | NM_000546.5 | exonic | stopgain SNV |
| COMET-0044M1-1 | APC | p.E1374X | c.G4120T | Chr5:g.112175411:G>T | NM_000038.4 | exonic | stopgain SNV |
|  | KRAS | p.G12C | c.G34T | Chr12:g.25398285:C>A | NM_033360.2 | exonic | nonsynonymous SNV |
|  | TP53 | p.R273C | c.C817T | Chr17:g.7577121:G>A | NM_000546.5 | exonic | nonsynonymous SNV |
|  | SMAD4 | p.S357P | c.T1069C | Chr18:g.48591906:T>C | NM_005359.5 | exonic | nonsynonymous SNV |
| COMET-0046M1-1 | APC | p.R1114X | c.C3340T | Chr5:g.112174631:C>T | NM_000038.4 | exonic | stopgain SNV |
|  | APC | p.F1354fs | c.4060_4061del | Chr5:g.112175351:TT>- | NM_000038.4 | exonic | frameshift deletion |
|  | TP53 | p.R248W | c.C742T | Chr17:g.7577539:G>A | NM_000546.5 | exonic | nonsynonymous SNV |
| COMET-0047M1-1 | KRAS | p.A146V | c.C437T | Chr12:g.25378561:G>A | NM_033360.2 | exonic | nonsynonymous SNV |
|  | KRAS | p.G12A | c.G35C | Chr12:g.25398284:C>G | NM_033360.2 | exonic | nonsynonymous SNV |
|  | SMAD4 | p.G365V | c.G1094T | Chr18:g.48591931:G>T | NM_005359.5 | exonic | nonsynonymous SNV |
| COMET-0049M1-1 | APC | NA | c.4288_4289GA | Chr5:g.112175579:AC>GA | NM_000038.4 | exonic | nonframeshift_substitution |
|  | APC | p.M1431R | c.T4292G | Chr5:g.112175583:T>G | NM_000038.4 | exonic | nonsynonymous SNV |
|  | KRAS | p.G12S | c.G34A | Chr12:g.25398285:C>T | NM_033360.2 | exonic | nonsynonymous SNV |
|  | TP53 | p.C242S | c.G725C | Chr17:g.7577556:C>G | NM_000546.5 | exonic | nonsynonymous SNV |
| COMET-0051M1-1 | PIK3CA | p.E542K | c.G1624A | Chr3:g.178936082:G>A | NM_006218.2 | exonic | nonsynonymous SNV |
|  | APC | p.R1450X | c.C4348T | Chr5:g.112175639:C>T | NM_000038.4 | exonic | stopgain SNV |
|  | KRAS | p.G12A | c.G35C | Chr12:g.25398284:C>G | NM_033360.2 | exonic | nonsynonymous SNV |
|  | TP53 | p.G245D | c.G734A | Chr17:g.7577547:C>T | NM_000546.5 | exonic | nonsynonymous SNV |
| COMET-0052M1-1 | KRAS | p.G12D | c.G35A | Chr12:g.25398284:C>T | NM_033360.2 | exonic | nonsynonymous SNV |
| COMET-0053M1-1 | KRAS | p.G12S | c.G34A | Chr12:g.25398285:C>T | NM_033360.2 | exonic | nonsynonymous SNV |
|  | TP53 | p.Y205X | c.T615G | Chr17:g.7578234:A>C | NM_000546.5 | exonic | stopgain SNV |
| COMET-0054M1-1 | PIK3CA | p.Q546R | c.A1637G | Chr3:g.178936095:A>G | NM_006218.2 | exonic | nonsynonymous SNV |
|  | KRAS | p.A146T | c.G436A | Chr12:g.25378562:C>T | NM_033360.2 | exonic | nonsynonymous SNV |
| COMET-0057M1-1 | APC | p.R1450X | c.C4348T | Chr5:g.112175639:C>T | NM_000038.4 | exonic | stopgain SNV |
|  | KRAS | p.G12V | c.G35T | Chr12:g.25398284:C>A | NM_033360.2 | exonic | nonsynonymous SNV |
| COMET-0059M1-1 |  |  |  |  |  |  |  |
| COMET-0061M1-1 | ERBB4 | p.I924N | c.T2771A | Chr2:g.212288975:A>T | NM_005235.2 | exonic | nonsynonymous SNV |
|  | APC | p.Q1303X | c.C3907T | Chr5:g.112175198:C>T | NM_000038.4 | exonic | stopgain SNV |
|  | KRAS | p.G12V | c.G35T | Chr12:g.25398284:C>A | NM_033360.2 | exonic | nonsynonymous SNV |
|  | TP53 | p.R196X | c.C586T | Chr17:g.7578263:G>A | NM_000546.5 | exonic | stopgain SNV |
| COMET-0062M1-1 | APC | p.Q1367X | c.C4099T | Chr5:g.112175390:C>T | NM_000038.4 | exonic | stopgain SNV |
|  | TP53 | p.R196X | c.C586T | Chr17:g.7578263:G>A | NM_000546.5 | exonic | stopgain SNV |
|  | APC | p.Q1367X | c.C4099T | Chr5:g.112175390:C>T | NM_000038.4 | exonic | stopgain SNV |
| COMET-0062M2-1 | TP53 | p.R196X | c.C586T | Chr17:g.7578263:G>A | NM_000546.5 | exonic | stopgain SNV |
|  | NRAS | p.Q61K | c.C181A | Chr1:g.115256530:G>T | NM_002524.3 | exonic | nonsynonymous SNV |
|  | TP53 | p.R248Q | c.G743A | Chr17:g.7577538:C>T | NM_000546.5 | exonic | nonsynonymous SNV |
| COMET-0065M1-1 | PDGFRA | p.I834I | c.C2502A | Chr4:g.55152070:C>A | NM_006206.4 | exonic | synonymous SNV |
|  | APC | p.Q1367X | c.C4099T | Chr5:g.112175390:C>T | NM_000038.4 | exonic | stopgain SNV |
| COMET-0071M1-1 | BRAF | p.G469R | c.G1405C | Chr7:g.140481403:C>G | NM_004333.4 | exonic | nonsynonymous SNV |
|  | TP53 | p.D208A | c.A623C | Chr17:g.7578226:T>G | NM_000546.5 | exonic | nonsynonymous SNV |
|  | TP53 | p.R196X | c.C586T | Chr17:g.7578263:G>A | NM_000546.5 | exonic | stopgain SNV |
|  |  |  |  |  |  |  |  |
