# Supplementary material for: Molecular signatures reflecting microenvironmental metabolism and chemotherapy-induced immunogenic cell death in colorectal liver metastases
Source: Oncotarget. 2017 Jul 18;8(44):76290–304. doi: 10.18632/oncotarget.19350 (PMC5652706; doi:10.18632/oncotarget.19350)
Supplement: Supplementary file 3 [file oncotarget-08-76290-s003.docx]

**Supplementary table 3:** **Univariable Cox proportional hazard analysis of OS and DFS, including DEGs**

|  | **OS** |  | **DFS** |  |  |  |  |  |  |  |
| --- | --- | --- | --- | --- | --- | --- | --- | --- | --- | --- |
| **Variable(n)** | | **Months (95% CI)** | **HR** | **95% CI** | **p-Value** | **Months (95% CI)** | **HR** | **95% CI** | **p-Value** | **DEGs** |
| Age | |  |  |  |  |  |  |  |  | 0 |
|  | <68 (25) | 43 (38-48) | Ref |  |  | 23 (16-30) | Ref |  |  |  |
|  | >68 (21) | 41 (35-46) | 1.5 | 0.6 – 3.9 | 0.4 | 25 (17-33) | 1.1 | 0.6 – 2.4 | 0.7 |  |
| Clinical Risk Score | |  |  |  |  |  |  |  |  | 0 |
|  | N0 (18) | 44 (39-49) | Ref |  |  | 25 (16-34) | Ref |  |  |  |
|  | N+ (20) | 39 (33-45) | 1.9 | 0.7 – 5.2 | 0.2 | 24 (16-33) | 1.1 | 0.5 - 2.5 | 0.8 |  |
|  | Size <5 (34) | 43 (39-46) | Ref |  |  | 26 (20-33) | Ref |  |  |  |
|  | Size >5 (4) | 28 (14-42) | 3.0 | 0.9 – 10.7 | 0.85 | 13 (0-27) | 2.2 | 0.6 - 7.4 | 0.2 |  |
|  | DFS > 1 year (9) | 40 (34-47) | Ref |  |  | 30 (18-43) | Ref |  |  |  |
|  | DFS < 1 year (29) | 41 (37-46) | 1.4 | 0.5 – 4.0 | 0.5 | 23 (16-30) | 1.7 | 0.6 – 4.9 | 0.4 |  |
|  | CLM < 1 (23) | 43 (39-47) | Ref |  |  | 25 (17-33) | Ref |  |  |  |
|  | CLM > 1 (15) | 37 (30-44) | 1.3 | 0.5 – 3.4 | 0.6 | 23 (14-32) | 1.0 | 0.4 – 2.4 | 0.9 |  |
|  | CEA* |  |  |  |  |  |  |  |  |  |
| *TP53* | |  |  |  |  |  |  |  |  | 0 |
|  | wt (11) | 43 (36-51) | Ref |  |  | 23 (12–33) | Ref |  |  |  |
|  | mt (35) | 42 (37-45) | 1.6 | 0.5 – 5.6 | 0.4 | 25 (18-31) | 0.9 | 0.4 – 2.2 | 0.9 |  |
| *APC* | |  |  |  |  |  |  |  |  | 0 |
|  | wt (18) | 40 (33-47) | Ref |  |  | 28 (19-36) | Ref |  |  |  |
|  | mt (28) | 43 (40-47) | 1.1 | 0.4 – 3.0 | 0.8 | 22 (15-29) | 1.2 | 0.6 – 2.7 | 0.6 |  |
| *KRAS* | |  |  |  |  |  |  |  |  | 0 |
|  | wt (19) | 42 (39-46) | Ref |  |  | 27 (18-36) | Ref | 18-36 |  |  |
|  | mt (27) | 36 (19-52) | 1.0 | 0.4 – 2.7 | 0.9 | 22 (15-29) | 1.2 | 0.6 – 2.6 | 0.6 |  |
| *PIK3CA* | |  |  |  |  |  |  |  |  | 0 |
|  | wt (37) | 42 (37-45) | Ref |  |  | 23 (17-29) | Ref |  |  |  |
|  | mt (9) | 43 (37-50) | 0.4 | 0.1 – 1.9 | 0.3 | 29 (18-39) | 0.6 | 0.2 – 1.6 | 0.3 |  |
| *SRC* | |  |  |  |  |  |  |  |  | 0 |
|  | wt (33) |  | Ref |  |  |  | Ref |  |  |  |
|  | amp (5) |  | 0.04 | 0 – 13.0 | 0.3 |  | 0.8 | 0.2 – 2.6 | 0.7 |  |
