# Supplementary material for: Molecular signatures reflecting microenvironmental metabolism and chemotherapy-induced immunogenic cell death in colorectal liver metastases
Source: Oncotarget. 2017 Jul 18;8(44):76290–304. doi: 10.18632/oncotarget.19350 (PMC5652706; doi:10.18632/oncotarget.19350)
Supplement: Supplementary file 4 [file oncotarget-08-76290-s004.docx]

| **Data_ set 1** |  |
| --- | --- |
| **Variance analysis filtering results: 111 genes derived from gene expression variance filter (var>5) applied to the metastasis samples. 55 genes formed a gene subset cluster** | |
| **111 genes derived by variance filter (var>5)** | **55 gene subset cluster** |
| ACSM2B | ACSM2B |
| ADH1A | ADH1A |
| ADH1C | ADH1C |
| ADH4 | ADH4 |
| AFM | AFM |
| AGXT | AGXT |
| AHSG | AHSG |
| ALB | ALB |
| ALDOB | ALDOB |
| AMBP | AMBP |
| APCDD1 | APOA1 |
| APOA1 | APOA2 |
| APOA2 | APOC3 |
| APOC3 | APOH |
| APOH | ARG1 |
| ARG1 | ASGR2 |
| ASGR2 | C3P1 |
| C10orf99 | C8A |
| C3P1 | C8B |
| C8A | C9 |
| C8B | CFHR2 |
| C9 | CPS1 |
| CD86 | CYP2C9 |
| CFHR2 | CYP8B1 |
| CHP2 | F2 |
| CLDN2 | FGA |
| CPS1 | FGB |
| CXCL14 | FGG |
| CYP2C9 | FGL1 |
| CYP8B1 | FTCD |
| DEFA5 | GC |
| DEFA6 | GCGR |
| DMBT1 | HAMP |
| DUSP27 | HP |
| F2 | HPR |
| FAM3B | HPX |
| FGA | HRG |
| FGB | HULC |
| FGG | IGFBP1 |
| FGL1 | ITIH1 |
| FTCD | ITIH2 |
| FZD10 | ITIH3 |
| GC | ITIH4 |
| GCGR | KNG1 |
| GPR179 | MAT1A |
| GPR182 | ORM1 |
| GSTT1 | ORM2 |
| H19 | PLG |
| HAMP | PPP6R1 |
| HP | RBP4 |
| HPR | SAA1 |
| HPX | SERPINC1 |
| HRG | TF |
| HSPA1A | TTR |
| HULC | UGT2B4 |
| IGF2 |  |
| IGFBP1 |  |
| IGLL5 |  |
| ITIH1 |  |
| ITIH2 |  |
| ITIH3 |  |
| ITIH4 |  |
| KLK8 |  |
| KNG1 |  |
| KRT6B |  |
| LCN15 |  |
| LEFTY1 |  |
| LGALS2 |  |
| LOC100128098 |  |
| LOC100507378 |  |
| LOC100509100 |  |
| MAT1A |  |
| MUC17 |  |
| MYH7B |  |
| OLFM4 |  |
| ORM1 |  |
| ORM2 |  |
| PIGR |  |
| PLA2G2A |  |
| PLG |  |
| PPP6R1 |  |
| PRAC |  |
| PRSS56 |  |
| PSPHP1 |  |
| RBP4 |  |
| REG1A |  |
| REG3A |  |
| RPS4Y1 |  |
| RPS4Y2 |  |
| SAA1 |  |
| SERPINC1 |  |
| SNAR.B2 |  |
| SNAR.D |  |
| SNAR.F |  |
| SNAR.G2 |  |
| SNAR.H |  |
| SOX2 |  |
| SPINK4 |  |
| TF |  |
| TTR |  |
| UGT2B4 |  |
| WIF1 |  |
| XIST |  |
| XLOC_001699 |  |
| XLOC_004384 |  |
| XLOC_005341 |  |
| XLOC_008559 |  |
| XLOC_009382 |  |
| XLOC_012294 |  |
| XLOC_013940 |  |
| XLOC_l2_007770 |  |

| **Data_ set 2** |  |  |  |  |
| --- | --- | --- | --- | --- |
| Ingenuity Pathway Analysis Summary for 55 clustered genes derived from gene expression variance filter (var>5) applied to the metastasis samples | | | | |
|  |  |  |  |  |
| *Canonical Pathway Analysis^1^* |  |  |  |  |
| **Top Canonical Pathways** | **p-value** | **molecules** | | |
| Acute Phase Response Signaling | 8,20E-37 | HAMP,ITIH3,TTR,HPX,APOH,APOA2,AHSG,AMBP,F2,FGG,PLG,ALB,HP,APOA1,ITIH2,TF,ORM1,ITIH4,SAA1,ORM2,FGB,HRG,FGA,RBP4 | | |
| FXR/RXR Activation | 1,30E-33 | KNG1,TTR,HPX,UGT2B4,APOH,APOA2,AHSG,AMBP,CYP8B1,ALB,HPR,APOA1,ORM1,TF,ITIH4,SAA1,ORM2,FGA,GC,RBP4,APOC3 | | |
| LXR/RXR Activation | 8,20E-30 | KNG1,TTR,HPX,APOH,APOA2,AHSG,AMBP,ALB,HPR,APOA1,TF,ORM1,ITIH4,SAA1,ORM2,FGA,GC,RBP4,APOC3 | | |
| Coagulation System | 3,10E-12 | KNG1,PLG,SERPINC1,FGB,FGA,FGG,F2 | | |
| Intrinsic Prothrombin Activation Pathway | 7,60E-11 | KNG1,SERPINC1,FGB,FGA,FGG,F2 | | |
| Extrinsic Prothrombin Activation Pathway | 3,90E-10 | SERPINC1,FGB,FGA,FGG,F2 | | |
| Clathrin-mediated Endocytosis Signaling | 9,50E-10 | ALB,APOA1,TF,ORM1,APOA2,ORM2,F2,RBP4,APOC3 | | |
| Atherosclerosis Signaling | 2,60E-08 | ALB,APOA1,ORM1,APOA2,ORM2,RBP4,APOC3 | | |
| IL-12 Signaling and Production in Macrophages | 4,90E-08 | ALB,APOA1,ORM1,APOA2,ORM2,RBP4,APOC3 | | |
| Production of Nitric Oxide and Reactive Oxygen Species in Macrophages | 3,70E-07 | ALB,APOA1,ORM1,APOA2,ORM2,RBP4,APOC3 | | |
|  |  |  |  |  |
|  |  |  |  |  |
| *Functional Analysis of gene subset^2^* |  |  |  |  |
| **Category** | **Functions Annotation** | **p-value** | **# molecules** | **molecules** |
| Lipid Metabolism, Small Molecule Biochemistry, Vitamin and Mineral Metabolism | metabolism of terpenoid | 3,24E-13 | 13 | ADH1C,ADH4,APOA1,APOA2,CYP2C9,CYP8B1,FGL1,GC,PLG,RBP4,SAA1,TTR,UGT2B4 |
| Cancer, Gastrointestinal Disease, Hepatic System Disease, Organismal Injury and Abnormalities | cholangiocarcinoma | 4,44E-13 | 8 | ADH1C,ALB,AMBP,FGA,GC,HPX,KNG1,SERPINC1 |
| Hematological System Development and Function | fibrinolysis | 1,66E-12 | 6 | APOH,F2,FGA,FGB,FGG,PLG |
| Metabolic Disease | amyloidosis | 1,76E-12 | 17 | AFM,ALB,APOA1,APOA2,APOC3,ARG1,FGA,GC,HP,HPX,HRG,KNG1,PLG,SAA1,SERPINC1,TF,TTR |
| Hematological System Development and Function | hemostasis | 4,97E-12 | 11 | APOH,ASGR2,CYP2C9,F2,FGA,FGB,FGG,HRG,KNG1,PLG,SERPINC1 |
| Organismal Injury and Abnormalities | fibrin clot | 1,11E-11 | 6 | APOH,F2,FGA,FGB,FGG,PLG |
| Hematological System Development and Function, Organismal Functions | coagulation of blood | 1,94E-11 | 10 | APOH,ASGR2,CYP2C9,F2,FGA,FGG,HRG,KNG1,PLG,SERPINC1 |
| Metabolic Disease, Neurological Disease, Psychological Disorders | Alzheimer's disease | 1,31E-10 | 15 | AFM,ALB,APOA1,APOA2,APOC3,ARG1,GC,HP,HPX,HRG,KNG1,PLG,SERPINC1,TF,TTR |
| Organismal Injury and Abnormalities | thrombus | 5,63E-10 | 7 | APOH,F2,FGA,FGB,FGG,PLG,SERPINC1 |
| Lipid Metabolism, Molecular Transport, Small Molecule Biochemistry | concentration of lipid | 1,68E-09 | 16 | ADH1C,AHSG,ALB,APOA1,APOA2,APOC3,CYP8B1,F2,FGL1,GC,GCGR,PLG,RBP4,SAA1,SERPINC1,TTR |
| Lipid Metabolism, Molecular Transport, Small Molecule Biochemistry | release of cholesterol | 4,80E-09 | 4 | APOA1,APOA2,PLG,SAA1 |
| Lipid Metabolism, Molecular Transport, Small Molecule Biochemistry, Vitamin and Mineral Metabolism | quantity of retinoid | 8,57E-09 | 5 | ADH1C,APOA1,APOC3,RBP4,TTR |
| Lipid Metabolism, Small Molecule Biochemistry, Vitamin and Mineral Metabolism | steroid metabolism | 9,63E-09 | 9 | APOA1,APOA2,CYP2C9,CYP8B1,FGL1,GC,PLG,SAA1,UGT2B4 |
| Vitamin and Mineral Metabolism | quantity of vitamin | 3,16E-08 | 6 | ADH1C,APOA1,APOC3,GC,RBP4,TTR |
| Molecular Transport | quantity of metal | 4,37E-08 | 11 | APOC3,F2,GCGR,HP,HPX,KNG1,ORM1,PLG,SAA1,TF,TTR |
|  |  |  |  |  |
|  |  |  |  |  |
| ^1^Canonical pathway analysis identified from the Ingenuity Knowledge base that were most significant to the gene set. | | | |  |
| ^2^Top ranked biological functions that were most significant to the genes eligible for analysis using a right tailed Fisher's exact test. | | | | |
| ^3^Upstream regulator analysis based on the 55 genes using Ingenuity Knowledge base. Top ranked regulators according to activation/inhibition z-score are displayed. The z-score is based on relationships between experimentally observed gene expression and function annotation data, as derived from the information compiled in the Ingenuity Knowledge Base. These relationships are associated with a direction of change that is either activating (z-score ≥ 2) or inhibiting (z-score ≤ -2). For activated upstream regulators predicted in this analysis, the top 15 regulators are displayed. | | | | |
|  |  |  |  |  |

| **Data_ set 3** |  |  |  |  |  |  |
| --- | --- | --- | --- | --- | --- | --- |
| Limma analysis results: 34 genes differentially expressed between *TP53* doublehit (both mt*TP53* and *TP53* deletion) (n=21) versus wt*TP53* or singlehit (n=17) patient samples | | | | | | |
| Gene | logFC | AveExpr | t | P.Value | adj.P.Val | B |
| CSGALNACT1 | -1,854 | 7,975 | -4,751 | 1,84E-05 | 0,069099 | 2,681 |
| CHP2 | 2,770 | 9,713 | 4,334 | 7,35E-05 | 0,069099 | 1,480 |
| SOSTDC1 | -1,403 | 6,051 | -4,281 | 8,73E-05 | 0,069099 | 1,331 |
| FOXL1 | -1,027 | 7,808 | -4,272 | 9,01E-05 | 0,069099 | 1,303 |
| LOC100507233 | 1,144 | 7,245 | 4,232 | 0,000102 | 0,069099 | 1,193 |
| TANC2 | -1,032 | 6,558 | -4,198 | 0,000114 | 0,069099 | 1,096 |
| MS4A8B | 1,634 | 6,766 | 4,180 | 0,000121 | 0,069099 | 1,047 |
| STYK1 | 1,146 | 8,162 | 4,136 | 0,00014 | 0,069099 | 0,925 |
| STRA6 | -1,719 | 7,799 | -4,122 | 0,000146 | 0,069099 | 0,885 |
| CMTM3 | -1,253 | 9,838 | -4,119 | 0,000147 | 0,069099 | 0,878 |
| MFAP2 | -1,314 | 7,154 | -4,080 | 0,000167 | 0,069099 | 0,770 |
| TRIM31 | 1,652 | 10,966 | 4,030 | 0,000196 | 0,069099 | 0,631 |
| TET1 | -0,893 | 6,589 | -4,028 | 0,000197 | 0,069099 | 0,625 |
| HOMER3 | -0,901 | 10,214 | -4,015 | 0,000206 | 0,069099 | 0,590 |
| LOC375295 | -1,313 | 5,990 | -3,958 | 0,000247 | 0,069099 | 0,433 |
| EMID1 | -1,007 | 6,490 | -3,943 | 0,000258 | 0,069099 | 0,394 |
| PDGFC | -1,250 | 7,706 | -3,942 | 0,000259 | 0,069099 | 0,390 |
| TMEM158 | -1,311 | 6,969 | -3,939 | 0,000262 | 0,069099 | 0,382 |
| GPC1 | -1,127 | 9,537 | -3,936 | 0,000264 | 0,069099 | 0,373 |
| ARL4C | -1,238 | 9,988 | -3,916 | 0,000281 | 0,069802 | 0,320 |
| SMO | -1,150 | 7,850 | -3,900 | 0,000296 | 0,069918 | 0,276 |
| XLOC_l2_010063 | 1,280 | 6,984 | 3,840 | 0,000357 | 0,080555 | 0,114 |
| CNIH3 | -1,066 | 6,271 | -3,800 | 0,000404 | 0,08545 | 0,007 |
| CLDN2 | 2,318 | 10,578 | 3,779 | 0,000431 | 0,08545 | -0,048 |
| NES | -0,933 | 8,471 | -3,773 | 0,00044 | 0,08545 | -0,066 |
| NFE2 | -1,913 | 7,133 | -3,762 | 0,000455 | 0,08545 | -0,095 |
| VIPR1 | 1,242 | 8,226 | 3,750 | 0,000471 | 0,08545 | -0,126 |
| AKR7L | 0,990 | 10,480 | 3,743 | 0,000482 | 0,08545 | -0,145 |
| OBSL1 | -1,119 | 7,582 | -3,731 | 0,0005 | 0,085681 | -0,177 |
| TMEM121 | -0,879 | 7,259 | -3,703 | 0,000546 | 0,088874 | -0,252 |
| UCN2 | -0,990 | 6,063 | -3,694 | 0,00056 | 0,088874 | -0,275 |
| FJX1 | -1,136 | 8,503 | -3,687 | 0,000573 | 0,088874 | -0,293 |
| XLOC_006277 | 1,735 | 8,755 | 3,640 | 0,000661 | 0,099489 | -0,417 |
| APCDD1 | -2,485 | 9,992 | -3,629 | 0,000684 | 0,099912 | -0,446 |

| **Data_ set 4** | |  |  |  |  |  |  |
| --- | --- | --- | --- | --- | --- | --- | --- |
| Limma analysis results: 44 genes differentially expressed between mt*NRAS* (n=4) versus wt*NRAS* (n=34) patient samples | | | | | | | |
| Gene | logFC | AveExpr | t | P.Value | adj.P.Val | B |  |
| CA1 | 2,597 | 5,657 | 6,736 | 1,77E-08 | 8,77E-05 | 8,751 |  |
| CASP1 | 2,983 | 9,178 | 5,475 | 1,52E-06 | 0,0038 | 4,885 |  |
| DDX60 | 2,216 | 7,958 | 4,957 | 9,10E-06 | 0,0144 | 3,323 |  |
| CA4 | 2,721 | 5,582 | 4,887 | 1,16E-05 | 0,0144 | 3,114 |  |
| CARD16 | 2,011 | 8,279 | 4,476 | 4,59E-05 | 0,0429 | 1,908 |  |
| MS4A8B | 2,745 | 6,766 | 4,395 | 6,00E-05 | 0,0429 | 1,674 |  |
| CLDN1 | -2,362 | 9,673 | -4,393 | 6,05E-05 | 0,0429 | 1,667 |  |
| DUOX1 | 1,640 | 5,846 | 4,272 | 8,99E-05 | 0,0558 | 1,321 |  |
| HRASLS2 | 2,517 | 7,119 | 4,164 | 0,000127 | 0,0628 | 1,017 |  |
| SI | 2,588 | 5,715 | 4,110 | 0,000151 | 0,0628 | 0,866 |  |
| NAPRT1 | 1,624 | 8,276 | 4,093 | 0,00016 | 0,0628 | 0,817 |  |
| IFIT3 | 2,228 | 8,121 | 4,060 | 0,000178 | 0,0628 | 0,726 |  |
| C2orf88 | 1,577 | 5,727 | 4,041 | 0,000189 | 0,0628 | 0,672 |  |
| IL17C | 1,656 | 5,320 | 4,031 | 0,000195 | 0,0628 | 0,645 |  |
| ABCA3 | 2,184 | 6,894 | 4,021 | 0,000201 | 0,0628 | 0,618 |  |
| IFI44L | 3,100 | 7,591 | 4,020 | 0,000202 | 0,0628 | 0,614 |  |
| GRAMD1B | 1,669 | 5,908 | 3,997 | 0,000217 | 0,0628 | 0,551 |  |
| IFIT1 | 2,899 | 8,664 | 3,983 | 0,000227 | 0,0628 | 0,512 |  |
| IRF9 | 1,454 | 9,915 | 3,949 | 0,000253 | 0,0648 | 0,420 |  |
| SERTAD4 | 1,499 | 5,328 | 3,931 | 0,000268 | 0,0648 | 0,369 |  |
| TMEM38A | 1,519 | 7,038 | 3,924 | 0,000274 | 0,0648 | 0,350 |  |
| TRIM7 | 2,022 | 6,109 | 3,896 | 0,000299 | 0,0673 | 0,275 |  |
| BTNL8 | 2,172 | 7,079 | 3,883 | 0,000312 | 0,0673 | 0,238 |  |
| CTSE | 2,598 | 6,956 | 3,813 | 0,000388 | 0,0755 | 0,047 |  |
| GCNT3 | 2,362 | 6,964 | 3,803 | 0,0004 | 0,0755 | 0,020 |  |
| C17orf78 | 2,538 | 5,782 | 3,789 | 0,000417 | 0,0755 | -0,016 |  |
| TSL | 2,765 | 6,230 | 3,787 | 0,00042 | 0,0755 | -0,022 |  |
| PRSS21 | 2,156 | 5,869 | 3,783 | 0,000426 | 0,0755 | -0,034 |  |
| XLOC_014019 | 1,408 | 4,873 | 3,745 | 0,000478 | 0,0819 | -0,135 |  |
| C6orf105 | 1,753 | 6,263 | 3,719 | 0,000518 | 0,0826 | -0,205 |  |
| SP110 | 1,349 | 9,362 | 3,704 | 0,000542 | 0,0826 | -0,243 |  |
| PLEKHB1 | -2,659 | 10,888 | -3,702 | 0,000545 | 0,0826 | -0,248 |  |
| PNMA1 | 1,434 | 8,346 | 3,700 | 0,000549 | 0,0826 | -0,254 |  |
| SNAR.D | -4,034 | 16,045 | -3,667 | 0,000607 | 0,0887 | -0,342 |  |
| SNAR.B2 | -3,857 | 16,411 | -3,657 | 0,000627 | 0,0889 | -0,369 |  |
| TESC | -2,374 | 13,975 | -3,606 | 0,000733 | 0,0994 | -0,504 |  |
| GPR158 | 1,636 | 5,371 | 3,600 | 0,000746 | 0,0994 | -0,520 |  |
| LACTB2 | 1,651 | 9,694 | 3,588 | 0,000772 | 0,0994 | -0,550 |  |
| RTP4 | 1,994 | 9,162 | 3,584 | 0,000781 | 0,0994 | -0,559 |  |
| ASB9 | -1,705 | 8,658 | -3,576 | 0,000801 | 0,0994 | -0,581 |  |
| LOC284577 | 1,531 | 4,958 | 3,554 | 0,000856 | 0,0997 | -0,639 |  |
| PARP9 | 1,343 | 8,467 | 3,551 | 0,000863 | 0,0997 | -0,646 |  |
| XLOC_000856 | -1,894 | 6,676 | -3,550 | 0,000868 | 0,0997 | -0,650 |  |
| SNAR.G2 | -4,224 | 15,321 | -3,544 | 0,000883 | 0,0997 | -0,666 |  |

| **Data_ set 5** |  |  |  |  |  |  |
| --- | --- | --- | --- | --- | --- | --- |
| Ingenuity Pathway Analysis - Upstream analysis based on 44 genes differentially expressed between mt*NRAS* (n=4) versus wt*NRAS* (n=34) patient samples | | | | | | |
|  |  |  |  |  |  |  |
| *Upstream Analysis^1^* |  |  |  |  |  |  |
| **Upstream Regulator** | **Molecule Type** | **Predicted Activation State** | **Activation z-score** | **p-value of overlap** | **Target molecules in dataset** | |
| IFNG | cytokine | Activated | 2 762 | 1,06E-05 | CASP1,GPR158,IFI44L,IFIT1,IFIT3,IRF9,RTP4,SP110 | |
| IFNL1 | cytokine | Activated | 2 433 | 5,66E-10 | DDX60,IFI44L,IFIT1,IFIT3,IRF9,SP110 | |
| TGM2 | enzyme | Activated | 2 433 | 1,38E-06 | DDX60,IFIT1,IFIT3,IRF9,PARP9,SP110 | |
| IFNA2 | cytokine | Activated | 2 385 | 5,16E-09 | DDX60,IFI44L,IFIT1,IFIT3,IRF9,SP110 | |
| PRL | cytokine | Activated | 2 236 | 2,88E-07 | IFI44L,IFIT1,IFIT3,IRF9,SP110 | |
| STAT1 | transcription regulator | Activated | 2 163 | 5,34E-07 | CASP1,IFIT1,IFIT3,IRF9,PARP9,SP110 | |
| TLR7 | transmembrane receptor | Activated | 2 000 | 2,81E-05 | IFI44L,IFIT1,IFIT3,IRF9 | |
| TLR9 | transmembrane receptor |  | 1 982 | 2,08E-04 | IFI44L,IFIT1,IFIT3,IRF9 | |
| IFNB1 | cytokine |  | 1 969 | 1,56E-04 | CASP1,IFIT1,IFIT3,IRF9 | |
| MAPK1 | kinase |  | -1 633 | 3,62E-06 | GRAMD1B,HRASLS2,IFIT1,IFIT3,IRF9,SP110 | |
| IL1RN | cytokine | Inhibited | -2 000 | 1,19E-05 | IFI44L,IFIT3,IRF9,RTP4 | |
| BTK | kinase | Inhibited | -2 000 | 1,19E-05 | IFI44L,IFIT1,IFIT3,IRF9 | |
| TRIM24 | transcription regulator | Inhibited | -2 000 | 1,76E-05 | DDX60,IFIT3,IRF9,RTP4 | |
| RARA | ligand-dependent nuclear receptor | Inhibited | -2 000 | 3,41E-04 | GPR158,IFI44L,RTP4,SP110 | |
| ESR1 | ligand-dependent nuclear receptor | Inhibited | -2 000 | 7,13E-04 | ABCA3,GPR158,IFI44L,RTP4,SP110 | |
|  |  |  |  |  |  |  |
|  |  |  |  |  |  |  |
| ^1^Upstream regulator analysis based on the 44 genes using Ingenuity Knowledge base. Top ranked regulators according to activation/inhibition z-score are displayed. The z-score is based on relationships between experimentally observed gene expression and function annotation data, as derived from the information compiled in the Ingenuity Knowledge Base. These relationships are associated with a direction of change that is either activating (z-score ≥ 2) or inhibiting (z-score ≤ -2). For activated upstream regulators predicted in this analysis, the top 15 regulators are displayed. | | | | | | |

| **Data_ set 6** | |  |  |  |  |  |  |
| --- | --- | --- | --- | --- | --- | --- | --- |
| Limma analysis results: 22 genes differentially expressed between mt*SMAD4* (n=7) versus wt*SMAD4* (n=31) patient samples | | | | | | | |
| Gene | logFC | AveExpr | t | P.Value | adj.P.Val | B |  |
| USP18 | 1,662 | 7,431 | 4,478 | 4,58E-05 | 0,06576 | 1,873 |  |
| GPR126 | 1,803 | 7,133 | 4,426 | 5,44E-05 | 0,06576 | 1,725 |  |
| HOXA7 | -2,696 | 10,634 | -4,296 | 8,33E-05 | 0,06576 | 1,357 |  |
| IFIT1 | 2,419 | 8,664 | 4,273 | 8,97E-05 | 0,06576 | 1,294 |  |
| TFCP2L1 | -1,953 | 9,794 | -4,261 | 9,35E-05 | 0,06576 | 1,259 |  |
| TRNP1 | 1,749 | 6,820 | 4,235 | 0,00010 | 0,06576 | 1,187 |  |
| PRSS21 | 1,854 | 5,869 | 4,221 | 0,00011 | 0,06576 | 1,147 |  |
| XLOC_011376 | -1,428 | 7,077 | -4,196 | 0,00012 | 0,06576 | 1,078 |  |
| TRIB1 | 1,156 | 13,680 | 4,186 | 0,00012 | 0,06576 | 1,050 |  |
| AGR3 | 2,875 | 9,193 | 4,129 | 0,00014 | 0,07108 | 0,892 |  |
| OAS2 | 1,352 | 7,198 | 4,054 | 0,00018 | 0,08201 | 0,687 |  |
| LOC100505912 | -1,434 | 8,375 | -3,994 | 0,00022 | 0,08918 | 0,522 |  |
| UCP2 | 1,576 | 7,763 | 3,957 | 0,00025 | 0,08918 | 0,422 |  |
| IRF7 | 1,171 | 11,913 | 3,902 | 0,00029 | 0,08918 | 0,274 |  |
| FOXQ1 | -1,676 | 13,380 | -3,896 | 0,00030 | 0,08918 | 0,257 |  |
| HRASLS2 | 1,890 | 7,119 | 3,878 | 0,00032 | 0,08918 | 0,210 |  |
| HOXA9 | -1,721 | 10,385 | -3,873 | 0,00032 | 0,08918 | 0,196 |  |
| IFI44L | 2,387 | 7,591 | 3,872 | 0,00032 | 0,08918 | 0,193 |  |
| LY6E | 2,119 | 9,184 | 3,802 | 0,00040 | 0,09398 | 0,007 |  |
| CCK | 1,801 | 5,785 | 3,801 | 0,00040 | 0,09398 | 0,004 |  |
| MX1 | 2,144 | 10,785 | 3,796 | 0,00041 | 0,09398 | -0,010 |  |
| CXXC4 | -1,554 | 7,048 | -3,791 | 0,00042 | 0,09398 | -0,024 |  |

| **Data_ set 7** | |  |  |  |  |  |
| --- | --- | --- | --- | --- | --- | --- |
| Limma analysis results: 59 genes differentially expressed between amp*ERBB2* (n=3) versus non-amp*ERBB2* (n=35) patient samples | | | | | | |
| Gene | logFC | AveExpr | t | P.Value | adj.P.Val | B |
| MED1 | 2,289 | 7,247 | 7,349 | 2,08E-09 | 1,03E-05 | 10,918 |
| ERBB2 | 3,019 | 9,580 | 7,071 | 5,56E-09 | 1,38E-05 | 10,045 |
| PGAP3 | 3,051 | 10,707 | 6,772 | 1,60E-08 | 2,65E-05 | 9,104 |
| CTAG1A | 3,545 | 5,723 | 5,879 | 3,76E-07 | 0,000467 | 6,277 |
| MIEN1 | 2,449 | 12,133 | 5,750 | 5,91E-07 | 0,000588 | 5,870 |
| GRB7 | 2,576 | 9,454 | 5,322 | 2,63E-06 | 0,002177 | 4,528 |
| TCAP | 2,058 | 7,639 | 5,208 | 3,90E-06 | 0,002771 | 4,172 |
| CTAG2 | 2,093 | 6,442 | 5,124 | 5,21E-06 | 0,003236 | 3,912 |
| ORMDL3 | 2,010 | 10,222 | 4,872 | 1,23E-05 | 0,006786 | 3,140 |
| ZNF655 | -2,455 | 8,678 | -4,771 | 1,73E-05 | 0,008588 | 2,833 |
| CCDC109B | -2,374 | 7,841 | -4,687 | 2,29E-05 | 0,009755 | 2,579 |
| ELOF1 | -1,816 | 8,879 | -4,679 | 2,36E-05 | 0,009755 | 2,555 |
| XLOC_012192 | 2,179 | 7,727 | 4,519 | 4,02E-05 | 0,015365 | 2,075 |
| PRKCH | -1,986 | 7,751 | -4,406 | 5,84E-05 | 0,020722 | 1,740 |
| YKT6 | -1,921 | 10,409 | -4,378 | 6,41E-05 | 0,021209 | 1,657 |
| ACTG1 | -1,734 | 14,262 | -4,353 | 6,96E-05 | 0,021591 | 1,583 |
| PPP1R1B | 3,304 | 10,677 | 4,330 | 7,49E-05 | 0,021873 | 1,517 |
| YPEL4 | 1,662 | 8,079 | 4,273 | 9,03E-05 | 0,023919 | 1,349 |
| TYRO3P | 1,708 | 5,334 | 4,269 | 9,15E-05 | 0,023919 | 1,337 |
| XLOC_005561 | 1,662 | 5,364 | 4,228 | 0,000104 | 0,024195 | 1,219 |
| ITGB5 | -2,035 | 9,765 | -4,226 | 0,000105 | 0,024195 | 1,213 |
| STARD3 | 1,917 | 11,731 | 4,220 | 0,000107 | 0,024195 | 1,195 |
| DNASE2 | -1,796 | 8,123 | -4,115 | 0,00015 | 0,032416 | 0,894 |
| TCTEX1D2 | -2,082 | 8,432 | -4,025 | 0,0002 | 0,039966 | 0,637 |
| XLOC_l2_012159 | 1,624 | 5,236 | 4,009 | 0,00021 | 0,039966 | 0,592 |
| TM9SF2 | -1,990 | 10,251 | -4,007 | 0,000212 | 0,039966 | 0,586 |
| XLOC_l2_001972 | 1,659 | 5,382 | 3,999 | 0,000217 | 0,039966 | 0,563 |
| SNORD83A | 2,180 | 8,314 | 3,905 | 0,000292 | 0,050472 | 0,299 |
| CAPN1 | -1,561 | 9,581 | -3,902 | 0,000295 | 0,050472 | 0,291 |
| COMTD1 | 1,666 | 11,778 | 3,889 | 0,000307 | 0,05083 | 0,254 |
| MED24 | 1,554 | 8,799 | 3,834 | 0,000365 | 0,058446 | 0,101 |
| MYH15 | 1,664 | 5,201 | 3,820 | 0,000381 | 0,05915 | 0,062 |
| XLOC_007222 | 1,951 | 8,304 | 3,794 | 0,000413 | 0,061367 | -0,011 |
| GPR108 | -1,523 | 8,629 | -3,786 | 0,000424 | 0,061367 | -0,033 |
| TNS3 | -1,762 | 9,909 | -3,779 | 0,000432 | 0,061367 | -0,051 |
| APEH | -1,648 | 9,172 | -3,753 | 0,000469 | 0,06477 | -0,124 |
| ZNF710 | 1,615 | 7,917 | 3,729 | 0,000505 | 0,066539 | -0,188 |
| NIPAL1 | 1,755 | 8,966 | 3,726 | 0,000509 | 0,066539 | -0,196 |
| RNU105A | 2,038 | 11,637 | 3,710 | 0,000536 | 0,068213 | -0,241 |
| XLOC_l2_015964 | 1,524 | 7,436 | 3,686 | 0,000576 | 0,070674 | -0,305 |
| FAM82B | 1,567 | 10,230 | 3,682 | 0,000583 | 0,070674 | -0,317 |
| PTPN20A | 1,584 | 5,335 | 3,632 | 0,000678 | 0,079332 | -0,451 |
| LAMC1 | -1,480 | 8,869 | -3,628 | 0,000687 | 0,079332 | -0,462 |
| LOC100287803 | 2,042 | 8,211 | 3,617 | 0,000711 | 0,079515 | -0,492 |
| NOXO1 | 2,261 | 8,006 | 3,613 | 0,00072 | 0,079515 | -0,505 |
| SNORD42B | 1,461 | 7,498 | 3,566 | 0,000831 | 0,088702 | -0,631 |
| C1QTNF1 | -1,439 | 7,075 | -3,555 | 0,000857 | 0,088702 | -0,659 |
| OR4N4 | 1,834 | 6,581 | 3,549 | 0,000874 | 0,088702 | -0,676 |
| EEF2 | -1,719 | 13,642 | -3,547 | 0,000878 | 0,088702 | -0,680 |
| RHOU | -1,550 | 8,743 | -3,541 | 0,000893 | 0,088702 | -0,695 |
| SLC35B1 | -1,454 | 9,635 | -3,531 | 0,00092 | 0,089625 | -0,722 |
| TRIM29 | -3,100 | 8,680 | -3,511 | 0,000978 | 0,093407 | -0,775 |
| OTUD1 | -1,693 | 8,509 | -3,496 | 0,001024 | 0,095664 | -0,816 |
| GAL3ST2 | 1,512 | 6,438 | 3,491 | 0,00104 | 0,095664 | -0,830 |
| LOC100506257 | 1,630 | 8,865 | 3,472 | 0,001098 | 0,097852 | -0,878 |
| PIK3R5 | 2,219 | 6,658 | 3,465 | 0,001122 | 0,097852 | -0,897 |
| ZNF581 | -1,601 | 7,861 | -3,460 | 0,001137 | 0,097852 | -0,909 |
| TEKT5 | 1,441 | 5,798 | 3,457 | 0,001148 | 0,097852 | -0,918 |
| XLOC_005368 | 1,432 | 5,272 | 3,453 | 0,001162 | 0,097852 | -0,928 |

| **Data_ set 8** | | |  |  |  |  |  |
| --- | --- | --- | --- | --- | --- | --- | --- |
| Limma analysis results: 36 genes differentially expressed between female (n=18) versus male (n=20) patient samples | | | | | | | |
| Gene | | logFC | AveExpr | t | P.Value | adj.P.Val | B |
| XIST | | 4,506 | 7,450 | 7,870 | 3,25E-10 | 1,62E-06 | 12,580 |
| RPS4Y1 | | -5,098 | 7,972 | -7,488 | 1,25E-09 | 2,70E-06 | 11,392 |
| RPS4Y2 | | -5,114 | 7,856 | -7,411 | 1,63E-09 | 2,70E-06 | 11,152 |
| XLOC_008015 | | 2,267 | 6,549 | 6,803 | 1,41E-08 | 1,61E-05 | 9,232 |
| XLOC_008185 | | 2,696 | 6,054 | 6,763 | 1,62E-08 | 1,61E-05 | 9,106 |
| DDX3Y | | -2,272 | 7,506 | -6,138 | 1,49E-07 | 0,000118 | 7,119 |
| TXLNG2P | | -2,571 | 6,636 | -6,106 | 1,67E-07 | 0,000118 | 7,017 |
| KDM5D | | -2,067 | 6,974 | -5,723 | 6,43E-07 | 0,000399 | 5,803 |
| TTTY15 | | -1,793 | 6,183 | -5,234 | 3,55E-06 | 0,001956 | 4,265 |
| LOC100509121 | | -2,835 | 6,834 | -5,115 | 5,34E-06 | 0,002651 | 3,896 |
| UTY | | -1,396 | 5,811 | -5,053 | 6,59E-06 | 0,002977 | 3,705 |
| XLOC_008323 | | -1,510 | 6,550 | -4,934 | 9,92E-06 | 0,004106 | 3,337 |
| FSCN1 | | 1,546 | 10,692 | 4,604 | 3,01E-05 | 0,011502 | 2,337 |
| NCRNA00185 | | -1,685 | 5,816 | -4,458 | 4,90E-05 | 0,017399 | 1,898 |
| CCDC88A | | 1,094 | 6,781 | 4,425 | 5,45E-05 | 0,018053 | 1,802 |
| USP9Y | | -1,313 | 5,855 | -4,320 | 7,71E-05 | 0,02393 | 1,491 |
| LOC100505908 | | -1,031 | 6,709 | -4,242 | 9,93E-05 | 0,029013 | 1,264 |
| ITGB7 | | 0,887 | 8,919 | 4,200 | 0,000114 | 0,031399 | 1,141 |
| C1QA | | 1,408 | 9,094 | 4,165 | 0,000127 | 0,032799 | 1,040 |
| LMCD1 | | 1,155 | 9,503 | 4,154 | 0,000132 | 0,032799 | 1,008 |
| EMP3 | | 1,130 | 8,337 | 4,099 | 0,000157 | 0,035988 | 0,851 |
| FAM129A | | 1,176 | 8,972 | 4,095 | 0,000159 | 0,035988 | 0,839 |
| MCM8 | | -0,909 | 9,236 | -3,845 | 0,000352 | 0,074703 | 0,132 |
| RBP1 | | 1,997 | 9,217 | 3,837 | 0,000361 | 0,074703 | 0,108 |
| ZFY | | -0,943 | 5,600 | -3,801 | 0,000403 | 0,080014 | 0,010 |
| RNF144B | | 0,838 | 7,020 | 3,736 | 0,000494 | 0,083 | -0,171 |
| C1QB | | 1,424 | 10,747 | 3,734 | 0,000496 | 0,083 | -0,176 |
| FLJ39095 | | -1,491 | 6,290 | -3,729 | 0,000504 | 0,083 | -0,190 |
| TAC3 | | -1,238 | 7,233 | -3,726 | 0,000508 | 0,083 | -0,197 |
| CCL3 | | 1,217 | 10,388 | 3,725 | 0,00051 | 0,083 | -0,200 |
| ANKRD35 | | 1,112 | 7,228 | 3,720 | 0,000518 | 0,083 | -0,214 |
| BASP1 | | 1,271 | 9,182 | 3,676 | 0,000593 | 0,090663 | -0,334 |
| LRRC25 | | 0,802 | 6,813 | 3,671 | 0,000602 | 0,090663 | -0,348 |
| IL4I1 | | 1,149 | 9,931 | 3,641 | 0,000659 | 0,093646 | -0,428 |
| RAB7B | | 0,906 | 6,649 | 3,641 | 0,00066 | 0,093646 | -0,429 |
| SLA | | 1,338 | 9,767432 | 3,614 | 0,000716 | 0,09875 | -0,502 |
| **Data_ set 9** | | |  |  |  |  |  |
| Limma analysis results: 50 genes differentially expressed between right-sided (n=10) versus left-sided (n=28) location of matched primary tumor | | | | | | | |
| Gene | logFC | | AveExpr | t | P.Value | adj.P.Val | B |
| PLAU | 1,471 | | 9,358 | 5,161 | 4,56E-06 | 0,022641 | 3,896 |
| XLOC_011331 | 1,382 | | 6,548 | 4,509 | 4,15E-05 | 0,073227 | 1,980 |
| TLCD1 | -1,371 | | 8,770 | -4,477 | 4,61E-05 | 0,073227 | 1,888 |
| C9orf53 | 1,810 | | 7,724 | 4,282 | 8,74E-05 | 0,073227 | 1,333 |
| CBS | -1,749 | | 10,942 | -4,254 | 9,57E-05 | 0,073227 | 1,254 |
| S100A2 | 1,939 | | 10,211 | 4,251 | 9,67E-05 | 0,073227 | 1,245 |
| PRDM1 | 1,232 | | 8,256 | 4,183 | 0,00012 | 0,073227 | 1,055 |
| TNFRSF6B | 1,543 | | 7,226 | 4,171 | 0,000125 | 0,073227 | 1,022 |
| PDPN | 1,820 | | 6,923 | 4,116 | 0,000149 | 0,073227 | 0,869 |
| LOC728034 | 1,260 | | 8,152 | 4,085 | 0,000165 | 0,073227 | 0,784 |
| AGPAT4.IT1 | 1,131 | | 6,689 | 4,079 | 0,000168 | 0,073227 | 0,766 |
| MMP14 | 1,159 | | 9,921 | 4,036 | 0,000193 | 0,073227 | 0,647 |
| CST1 | 2,033 | | 6,478 | 4,005 | 0,000213 | 0,073227 | 0,562 |
| CST2 | 1,703 | | 6,431 | 3,978 | 0,000232 | 0,073227 | 0,487 |
| FAM155A | 1,495 | | 6,423 | 3,974 | 0,000235 | 0,073227 | 0,477 |
| RTEL1 | 1,219 | | 10,263 | 3,972 | 0,000236 | 0,073227 | 0,473 |
| GJB2 | 1,480 | | 9,159 | 3,934 | 0,000266 | 0,075893 | 0,368 |
| ADAM19 | 1,248 | | 7,901 | 3,921 | 0,000278 | 0,075893 | 0,332 |
| PRDM8 | 1,109 | | 6,109 | 3,903 | 0,000294 | 0,075893 | 0,284 |
| UNC13D | 1,466 | | 7,734 | 3,875 | 0,00032 | 0,075893 | 0,209 |
| F3 | 1,312 | | 8,900 | 3,871 | 0,000324 | 0,075893 | 0,197 |
| MTL5 | -0,938 | | 7,390 | -3,849 | 0,000348 | 0,075893 | 0,137 |
| SLC13A3 | -1,437 | | 7,705 | -3,846 | 0,000351 | 0,075893 | 0,128 |
| LAIR2 | 1,685 | | 6,418 | 3,831 | 0,000367 | 0,07601 | 0,090 |
| SPRR1B | 1,451 | | 6,070 | 3,806 | 0,000398 | 0,077394 | 0,022 |
| IMPDH2 | -0,976 | | 11,253 | -3,798 | 0,000407 | 0,077394 | 0,002 |
| MGC24103 | 1,286 | | 7,205 | 3,788 | 0,000421 | 0,077394 | -0,027 |
| MRC2 | 1,454 | | 11,358 | 3,753 | 0,000468 | 0,082125 | -0,118 |
| RRAS2 | -1,056 | | 9,443 | -3,724 | 0,000512 | 0,082125 | -0,196 |
| FAM203A | -1,043 | | 9,818 | -3,717 | 0,000523 | 0,082125 | -0,214 |
| SPRR3 | 1,339 | | 6,504 | 3,713 | 0,000529 | 0,082125 | -0,224 |
| IGANRP | 1,044 | | 6,444 | 3,713 | 0,000529 | 0,082125 | -0,224 |
| ADAM8 | 1,463 | | 11,863 | 3,690 | 0,000568 | 0,08544 | -0,285 |
| PDIA2 | -1,395 | | 6,703 | -3,669 | 0,000606 | 0,086754 | -0,341 |
| WDR86 | 1,070 | | 6,501 | 3,657 | 0,000628 | 0,086754 | -0,372 |
| WNT5A | 1,336 | | 7,302 | 3,655 | 0,000633 | 0,086754 | -0,378 |
| CARD6 | 1,090 | | 8,371 | 3,644 | 0,000654 | 0,086754 | -0,406 |
| KCNE3 | -1,108 | | 9,650 | -3,639 | 0,000664 | 0,086754 | -0,420 |
| MILR1 | 1,022 | | 6,921 | 3,629 | 0,000684 | 0,087057 | -0,445 |
| REEP1 | -1,302 | | 6,763 | -3,612 | 0,00072 | 0,089432 | -0,490 |
| ARL4C | 1,306 | | 9,988 | 3,590 | 0,000771 | 0,093413 | -0,549 |
| SLC25A29 | 0,965 | | 11,685 | 3,552 | 0,000865 | 0,099405 | -0,647 |
| DCBLD2 | 1,146 | | 8,006 | 3,529 | 0,000927 | 0,099405 | -0,707 |
| TMEM236 | -1,031 | | 6,026 | -3,523 | 0,000942 | 0,099405 | -0,721 |
| TNFAIP2 | 1,413 | | 11,951 | 3,517 | 0,000958 | 0,099405 | -0,735 |
| DSCC1 | -0,989 | | 7,312 | -3,517 | 0,000958 | 0,099405 | -0,735 |
| S100A4 | 1,814 | | 12,377 | 3,512 | 0,000975 | 0,099405 | -0,751 |
| LCMT2 | -0,946 | | 7,922 | -3,509 | 0,000983 | 0,099405 | -0,758 |
| BST2 | 1,815 | | 11,274 | 3,508 | 0,000987 | 0,099405 | -0,761 |
| QSOX1 | 0,939 | | 9,675 | 3,503 | 0,001001 | 0,099405 | -0,773 |

| **Data_ set 10** |  |  |  |  |  |  | |  |  |  |
| --- | --- | --- | --- | --- | --- | --- | --- | --- | --- | --- |
| Ingenuity Pathway Analysis Summary for 50 genes differentially expressed between right-sided (n=10) versus left-sided (n=28) location of matched primary tumor. Of 50 genes, 46 were eligble for IPA core analysis. | | | | | | | | | | |
|  |  | | | | | | | | | |
| *Canonical Pathway Analysis^1^* |  |  |  |  |  |  |  |  |  |  |
| **Top Canonical Pathways** | **p-value** | **z-score** | **molecules** | | | | | | | |
| Role of Tissue Factor in Cancer | 1,95E-03 | NaN | PDIA2,RRAS2,F3 | | | | | | | |
| Oncostatin M Signaling | 2,81E-03 | NaN | RRAS2,PLAU | | | | | | | |
| Coagulation System | 2,97E-03 | NaN | PLAU,F3 | | | | | | | |
| Cysteine Biosynthesis/Homocysteine Degradation | 4,63E-03 | NaN | CBS/CBSL | | | | | | | |
| Glioma Invasiveness Signaling | 7,72E-03 | NaN | RRAS2,PLAU | | | | | | | |
| Colorectal Cancer Metastasis Signaling | 1,63E-03 | NaN | RRAS2,MMP14,WNT5A | | | | | | | |
|  | | | | | | | | | | |
| *Functional Analysis of gene subset^2^* |  | | | | | | | | | |
| **Category** | **Functions Annotation** | **p-value** | **Predicted Activation State** | **Activation z-score** | **# molecules** | | **molecules** | | | |
| Organismal Functions, Organismal Injury and Abnormalities, Tissue Morphology | healing of wound | 4,51E-05 |  |  | 3 | DCBLD2,SPRR3,WNT5A | | | | |
| Gene Expression | binding of interferon-stimulated response element | 2,33E-04 |  |  | 2 | PLAU,PRDM1 | | | | |
| Cell Death and Survival | killing of tumor cell lines | 5,94E-04 |  |  | 3 | F3,MMP14,PLAU | | | | |
| Cellular Development, Cellular Growth and Proliferation, Embryonic Development, Organismal Development, Tissue Development | proliferation of mesenchymal cells | 7,84E-04 |  |  | 2 | S100A4,WNT5A | | | | |
| Cell-To-Cell Signaling and Interaction, Cellular Assembly and Organization | binding of cell surface | 8,75E-04 |  |  | 2 | MMP14,PDIA2 | | | | |
| Cellular Movement, Hematological System Development and Function, Immune Cell Trafficking | migration of mononuclear leukocytes | 9,26E-04 |  | 0,147 | 4 | MMP14,PLAU,TNFRSF6B,WNT5A | | | | |
| Inflammatory Response, Respiratory Disease | inflammation of lung | 1,00E-03 |  |  | 4 | IMPDH2,PLAU,RTEL1,S100A4 | | | | |
| Organismal Injury and Abnormalities | blood clot | 1,52E-03 |  |  | 2 | F3,PLAU | | | | |
| Cell-mediated Immune Response, Cellular Movement, Hematological System Development and Function, Immune Cell Trafficking | T cell migration | 1,79E-03 |  |  | 3 | PLAU,TNFRSF6B,WNT5A | | | | |
| Cancer, Organismal Injury and Abnormalities, Reproductive System Disease | breast cancer | 1,92E-03 |  |  | 14 | CST1,CST2,DSCC1,FAM155A,GJB2,MMP14,PDPN,PLAU,PRDM1,QSOX1,RRAS2,S100A2,S100A4,WNT5A | | | | |
| Cellular Movement, Hematological System Development and Function, Immune Cell Trafficking | cell movement of myeloid cells | 2,59E-03 |  | 1 046 | 4 | MMP14,PLAU,TNFRSF6B,WNT5A | | | | |
| Cell-To-Cell Signaling and Interaction | activation of cells | 3,37E-03 |  | 1 457 | 5 | F3,MMP14,PLAU,TNFRSF6B,WNT5A | | | | |
| Inflammatory Response | inflammation of organ | 6,42E-03 |  |  | 7 | F3,IMPDH2,PLAU,PRDM1,RTEL1,S100A2,S100A4 | | | | |
| Cellular Movement | migration of cells | 7,08E-03 |  | 1 487 | 9 | BST2,DCBLD2,F3,MMP14,PLAU,S100A2,S100A4,TNFRSF6B,WNT5A | | | | |
| Cellular Development, Cellular Growth and Proliferation | cell proliferation of breast cancer cell lines | 7,35E-03 | Increased | 2 075 | 5 | F3,MMP14,PLAU,S100A4,WNT5A | | | | |
|  |  | | | | | | | | | |
|  |  |  |  |  |  |  |  |  |  |  |
| *Upstream Analysis^3^* |  |  |  |  |  |  |  |  |  |  |
| **Upstream Regulator** | **Exp Log Ratio** | **Molecule Type** | **Predicted Activation State** | **Activation z-score** | **p-value of overlap** | **Target molecules in dataset** | | | | |
| TNF |  | cytokine | Activated | 2 959 | 3,79E-07 | ADAM8,BST2,F3,PDPN,PLAU,PRDM1,TNFAIP2,TNFRSF6B,WNT5A | | | | |
| TGFB1 |  | growth factor |  | 1 946 | 4,23E-03 | ADAM19,PLAU,S100A4,WNT5A | | | | |
| MGEA5 |  | enzyme |  | 1 000 | 1,31E-03 | ADAM19,MMP14,PLAU,S100A2 | | | | |
| TP63 |  | transcription regulator |  | 0,150 | 1,79E-07 | BST2,F3,PLAU,S100A2,S100A4,SPRR3,WNT5A | | | | |
|  |  |  |  | | | | | | | |
|  |  |  |  |  |  |  |  |  |  |  |
| ^1^Canonical pathway analysis identified from the Ingenuity Knowledge base that were most significant to the gene set. | | |  |  |  |  |  |  |  |  |
| ^2^Top ranked biological functions that were most significant to the genes eligible for analysis using a right tailed Fisher's exact test. | | | |  | | | | | | |
| ^3^Upstream regulator analysis based on the 50 genes using Ingenuity Knowledge base. Top ranked regulators according to activation/inhibition z-score are displayed. The z-score is based on relationships between experimentally observed gene expression and function annotation data, as derived from the information compiled in the Ingenuity Knowledge Base. These relationships are associated with a direction of change that is either activating (z-score ≥ 2) or inhibiting (z-score ≤ -2). For activated upstream regulators predicted in this analysis, the top 15 regulators are displayed. | | | | | | | | | | |

| **Data_ set 11** | |  |  |  |  |  |  |
| --- | --- | --- | --- | --- | --- | --- | --- |
| Limma analysis results: 208 genes differentially expressed between NACT treated (n=15) versus non-treated (n=29) metastatic samples | | | | | | | |
| Gene | logFC | AveExpr | t | P.Value | adj.P.Val | B |  |
| ACP5 | 1,375 | 12,454 | 4,999 | 7,62E-06 | 0,013485 | 3,533 |  |
| FAM13A | -1,459 | 9,237 | -4,980 | 8,14E-06 | 0,013485 | 3,474 |  |
| TPM2 | 1,224 | 12,448 | 4,841 | 1,31E-05 | 0,013485 | 3,051 |  |
| LAIR1 | 1,033 | 7,810 | 4,840 | 1,31E-05 | 0,013485 | 3,051 |  |
| CCDC88A | 1,133 | 6,781 | 4,830 | 1,36E-05 | 0,013485 | 3,020 |  |
| FABP6 | -1,869 | 9,170 | -4,732 | 1,90E-05 | 0,015715 | 2,722 |  |
| MS4A7 | 1,409 | 9,188 | 4,631 | 2,66E-05 | 0,017725 | 2,421 |  |
| MS4A4A | 1,538 | 8,401 | 4,608 | 2,88E-05 | 0,017725 | 2,350 |  |
| COL4A5 | 1,259 | 6,056 | 4,576 | 3,21E-05 | 0,017725 | 2,255 |  |
| SLA | 1,550 | 9,767 | 4,472 | 4,54E-05 | 0,022547 | 1,947 |  |
| C10orf99 | -2,668 | 11,725 | -4,418 | 5,44E-05 | 0,024548 | 1,787 |  |
| TREM2 | 1,131 | 6,517 | 4,388 | 6,00E-05 | 0,024817 | 1,700 |  |
| KLHL6 | 1,002 | 6,773 | 4,342 | 6,97E-05 | 0,026637 | 1,567 |  |
| C1orf38 | 0,953 | 7,468 | 4,267 | 8,93E-05 | 0,030265 | 1,347 |  |
| FAM129A | 1,194 | 8,972 | 4,260 | 9,14E-05 | 0,030265 | 1,326 |  |
| DOCK10 | 0,994 | 7,270 | 4,213 | 0,000106 | 0,033015 | 1,192 |  |
| DPEP2 | 0,898 | 6,064 | 4,171 | 0,000122 | 0,033922 | 1,069 |  |
| LAPTM5 | 1,420 | 8,519 | 4,141 | 0,000135 | 0,033922 | 0,984 |  |
| CSF1R | 1,268 | 10,650 | 4,121 | 0,000144 | 0,033922 | 0,926 |  |
| SLC1A3 | 1,009 | 6,553 | 4,118 | 0,000145 | 0,033922 | 0,919 |  |
| HLA.DMB | 1,431 | 10,063 | 4,089 | 0,000159 | 0,033922 | 0,837 |  |
| HLA.DPB2 | 1,126 | 8,206 | 4,086 | 0,00016 | 0,033922 | 0,829 |  |
| TFF3 | -1,302 | 14,036 | -4,085 | 0,000161 | 0,033922 | 0,825 |  |
| C1orf162 | 0,884 | 7,601 | 4,080 | 0,000164 | 0,033922 | 0,809 |  |
| CECR1 | 1,422 | 8,618 | 4,050 | 0,00018 | 0,03498 | 0,726 |  |
| ALOX15B | 1,005 | 7,600 | 4,045 | 0,000183 | 0,03498 | 0,711 |  |
| LST1 | 1,125 | 8,049 | 3,990 | 0,000218 | 0,040146 | 0,556 |  |
| COL8A2 | 0,900 | 6,193 | 3,976 | 0,000229 | 0,040548 | 0,515 |  |
| HLA.DPB1 | 1,230 | 9,105 | 3,962 | 0,000238 | 0,040815 | 0,478 |  |
| PLA2G7 | 1,217 | 8,099 | 3,950 | 0,000248 | 0,041012 | 0,444 |  |
| TYROBP | 1,247 | 12,209 | 3,913 | 0,000279 | 0,042076 | 0,340 |  |
| SH3RF2 | -0,917 | 8,359 | -3,894 | 0,000296 | 0,042076 | 0,288 |  |
| HOXB2 | 0,990 | 7,625 | 3,893 | 0,000297 | 0,042076 | 0,284 |  |
| VSIG4 | 1,451 | 9,239 | 3,871 | 0,000318 | 0,042076 | 0,222 |  |
| FERMT1 | -1,101 | 10,228 | -3,863 | 0,000326 | 0,042076 | 0,202 |  |
| C20orf194 | 0,896 | 7,087 | 3,859 | 0,00033 | 0,042076 | 0,190 |  |
| HLA.DPA1 | 1,368 | 10,549 | 3,859 | 0,000331 | 0,042076 | 0,189 |  |
| C1QB | 1,453 | 10,747 | 3,856 | 0,000334 | 0,042076 | 0,181 |  |
| MSR1 | 0,858 | 6,639 | 3,853 | 0,000337 | 0,042076 | 0,173 |  |
| SLITRK4 | 0,913 | 6,393 | 3,851 | 0,000339 | 0,042076 | 0,168 |  |
| MGC24103 | 1,124 | 7,205 | 3,841 | 0,00035 | 0,042107 | 0,139 |  |
| PLCB4 | -1,982 | 9,000 | -3,835 | 0,000356 | 0,042107 | 0,124 |  |
| PPP1R1B | -1,574 | 10,677 | -3,794 | 0,000404 | 0,04573 | 0,012 |  |
| SASH3 | 0,929 | 6,657 | 3,787 | 0,000414 | 0,04573 | -0,010 |  |
| EVL | 1,081 | 9,299 | 3,787 | 0,000414 | 0,04573 | -0,010 |  |
| MFAP5 | 0,990 | 6,868 | 3,777 | 0,000427 | 0,046136 | -0,037 |  |
| IL10RA | 1,255 | 9,373 | 3,748 | 0,000468 | 0,046958 | -0,116 |  |
| TLR7 | 0,830 | 5,928 | 3,741 | 0,000478 | 0,046958 | -0,135 |  |
| NEXN | 0,911 | 7,457 | 3,739 | 0,000481 | 0,046958 | -0,140 |  |
| FCGR1B | 0,952 | 7,283 | 3,738 | 0,000482 | 0,046958 | -0,144 |  |
| CD84 | 0,863 | 6,863 | 3,734 | 0,000488 | 0,046958 | -0,154 |  |
| SLC15A3 | 1,165 | 8,908 | 3,731 | 0,000492 | 0,046958 | -0,160 |  |
| SRGN | 1,355 | 9,714 | 3,724 | 0,000503 | 0,047107 | -0,180 |  |
| PPP1R14C | -1,328 | 7,671 | -3,709 | 0,000527 | 0,048507 | -0,222 |  |
| HCLS1 | 1,095 | 10,717 | 3,688 | 0,000562 | 0,048915 | -0,278 |  |
| FZD10 | 2,781 | 7,785 | 3,686 | 0,000565 | 0,048915 | -0,283 |  |
| HLA.DMA | 0,951 | 9,031 | 3,680 | 0,000575 | 0,048915 | -0,299 |  |
| MS4A6A | 0,943 | 6,858 | 3,680 | 0,000577 | 0,048915 | -0,300 |  |
| TLR1 | 0,855 | 6,590 | 3,674 | 0,000586 | 0,048915 | -0,315 |  |
| C16orf54 | 0,957 | 6,539 | 3,672 | 0,000591 | 0,048915 | -0,322 |  |
| C20orf103 | 1,030 | 6,733 | 3,662 | 0,000608 | 0,049136 | -0,347 |  |
| TM7SF4 | 0,897 | 6,299 | 3,660 | 0,000613 | 0,049136 | -0,355 |  |
| NCF2 | 1,020 | 7,689 | 3,640 | 0,000651 | 0,050147 | -0,407 |  |
| TSPAN8 | -0,904 | 13,925 | -3,637 | 0,000658 | 0,050147 | -0,416 |  |
| ENGASE | -0,776 | 8,912 | -3,626 | 0,000679 | 0,050147 | -0,444 |  |
| CD52 | 1,225 | 10,806 | 3,624 | 0,000684 | 0,050147 | -0,450 |  |
| HS3ST2 | 1,012 | 5,835 | 3,623 | 0,000686 | 0,050147 | -0,454 |  |
| GPNMB | 0,941 | 7,827 | 3,618 | 0,000696 | 0,050147 | -0,466 |  |
| RAB7B | 0,875 | 6,649 | 3,615 | 0,000702 | 0,050147 | -0,474 |  |
| LY86 | 0,849 | 8,658 | 3,610 | 0,000713 | 0,050147 | -0,486 |  |
| SLC31A2 | 0,980 | 8,376 | 3,609 | 0,000717 | 0,050147 | -0,492 |  |
| CYBB | 1,093 | 9,013 | 3,602 | 0,000732 | 0,050264 | -0,510 |  |
| RGS1 | 1,557 | 10,092 | 3,599 | 0,000739 | 0,050264 | -0,518 |  |
| PCDHGA2 | -1,138 | 9,117 | -3,592 | 0,000753 | 0,050558 | -0,535 |  |
| SPI1 | 0,917 | 8,723 | 3,587 | 0,000765 | 0,050688 | -0,549 |  |
| SFRP4 | 0,975 | 6,307 | 3,564 | 0,00082 | 0,05358 | -0,610 |  |
| SLAMF8 | 1,171 | 9,021 | 3,555 | 0,000843 | 0,0539 | -0,634 |  |
| HLA.DRA | 1,731 | 10,255 | 3,554 | 0,000846 | 0,0539 | -0,638 |  |
| RAB34 | 0,900 | 6,559 | 3,541 | 0,00088 | 0,055353 | -0,672 |  |
| UCHL1 | 1,734 | 8,172 | 3,529 | 0,000911 | 0,056552 | -0,702 |  |
| PCK1 | -1,998 | 10,590 | -3,522 | 0,00093 | 0,057043 | -0,720 |  |
| MAFB | 1,135 | 10,481 | 3,490 | 0,001026 | 0,061544 | -0,806 |  |
| TNFSF13B | 0,880 | 8,692 | 3,489 | 0,001028 | 0,061544 | -0,808 |  |
| HLA.DRB6 | 0,837 | 5,542 | 3,484 | 0,001045 | 0,061806 | -0,822 |  |
| GIMAP8 | 0,745 | 6,335 | 3,474 | 0,001075 | 0,062825 | -0,847 |  |
| PECAM1 | 0,860 | 7,843 | 3,468 | 0,001096 | 0,063236 | -0,864 |  |
| TRPV2 | 0,922 | 9,384 | 3,464 | 0,001108 | 0,063236 | -0,873 |  |
| NOXO1 | -1,185 | 8,006 | -3,455 | 0,001137 | 0,063609 | -0,896 |  |
| LEFTY1 | -2,907 | 9,954 | -3,452 | 0,001148 | 0,063609 | -0,905 |  |
| MILR1 | 0,842 | 6,921 | 3,451 | 0,001153 | 0,063609 | -0,908 |  |
| C1QA | 1,201 | 9,094 | 3,439 | 0,001194 | 0,065139 | -0,939 |  |
| ANKRD35 | 1,018 | 7,228 | 3,436 | 0,001207 | 0,065139 | -0,948 |  |
| FLJ31485 | 0,828 | 5,887 | 3,419 | 0,001266 | 0,067295 | -0,990 |  |
| CMKLR1 | 0,844 | 7,049 | 3,417 | 0,001274 | 0,067295 | -0,995 |  |
| GPCPD1 | -0,996 | 11,889 | -3,383 | 0,001411 | 0,072014 | -1,085 |  |
| SGK1 | 1,044 | 10,096 | 3,382 | 0,001415 | 0,072014 | -1,087 |  |
| XLOC_001085 | -1,001 | 6,070 | -3,379 | 0,001426 | 0,072014 | -1,094 |  |
| FAM49A | 0,804 | 6,762 | 3,379 | 0,001428 | 0,072014 | -1,095 |  |
| C2orf89 | -0,928 | 10,226 | -3,377 | 0,001435 | 0,072014 | -1,100 |  |
| ANKRD37 | -1,181 | 10,140 | -3,372 | 0,001455 | 0,072085 | -1,111 |  |
| PCDHGA12 | -0,938 | 7,324 | -3,370 | 0,001466 | 0,072085 | -1,118 |  |
| VMO1 | 0,869 | 7,182 | 3,359 | 0,001513 | 0,072999 | -1,145 |  |
| TLR2 | 0,968 | 8,341 | 3,359 | 0,001514 | 0,072999 | -1,146 |  |
| HTRA4 | 0,890 | 6,247 | 3,341 | 0,001595 | 0,076182 | -1,192 |  |
| ALOX5AP | 0,945 | 7,131 | 3,334 | 0,001627 | 0,076975 | -1,209 |  |
| KIF5C | 0,933 | 5,756 | 3,327 | 0,001661 | 0,077587 | -1,227 |  |
| FAM78A | 0,748 | 6,784 | 3,319 | 0,001703 | 0,077587 | -1,249 |  |
| APBB1IP | 0,879 | 7,508 | 3,314 | 0,001728 | 0,077587 | -1,261 |  |
| XLOC_009167 | 0,716 | 5,654 | 3,308 | 0,001756 | 0,077587 | -1,276 |  |
| LCP1 | 1,149 | 9,642 | 3,305 | 0,001774 | 0,077587 | -1,284 |  |
| IGFBP6 | 0,975 | 6,543 | 3,304 | 0,001777 | 0,077587 | -1,286 |  |
| KCTD12 | 1,290 | 10,086 | 3,303 | 0,001783 | 0,077587 | -1,289 |  |
| EHF | -1,113 | 11,730 | -3,303 | 0,001784 | 0,077587 | -1,289 |  |
| PLEK | 0,803 | 6,667 | 3,301 | 0,001792 | 0,077587 | -1,293 |  |
| LIPA | 0,860 | 11,019 | 3,295 | 0,001825 | 0,077587 | -1,309 |  |
| HAVCR2 | 0,744 | 6,410 | 3,290 | 0,001849 | 0,077587 | -1,320 |  |
| C1orf54 | 0,827 | 11,053 | 3,290 | 0,001849 | 0,077587 | -1,320 |  |
| MRVI1 | 1,064 | 8,770 | 3,289 | 0,001857 | 0,077587 | -1,324 |  |
| CH25H | 1,154 | 6,874 | 3,289 | 0,001859 | 0,077587 | -1,325 |  |
| PIGR | -3,144 | 11,727 | -3,274 | 0,001937 | 0,077648 | -1,361 |  |
| XLOC_001788 | 0,710 | 6,585 | 3,274 | 0,001939 | 0,077648 | -1,362 |  |
| TUBB6 | 1,006 | 10,980 | 3,271 | 0,001956 | 0,077648 | -1,369 |  |
| SNX32 | 0,745 | 7,036 | 3,270 | 0,001963 | 0,077648 | -1,372 |  |
| VCAN | 1,067 | 10,204 | 3,270 | 0,001963 | 0,077648 | -1,373 |  |
| BCAT1 | 0,836 | 6,813 | 3,268 | 0,001976 | 0,077648 | -1,378 |  |
| EMP3 | 0,924 | 8,337 | 3,267 | 0,001977 | 0,077648 | -1,379 |  |
| FES | 0,727 | 6,767 | 3,265 | 0,001989 | 0,077648 | -1,384 |  |
| MYL9 | 0,958 | 8,883 | 3,263 | 0,002005 | 0,077648 | -1,391 |  |
| NFAM1 | 0,852 | 7,603 | 3,252 | 0,002069 | 0,077648 | -1,418 |  |
| PLCG2 | 0,803 | 6,833 | 3,249 | 0,002085 | 0,077648 | -1,425 |  |
| ARHGAP10 | 0,707 | 6,646 | 3,248 | 0,00209 | 0,077648 | -1,427 |  |
| SPARCL1 | 1,276 | 7,853 | 3,246 | 0,002102 | 0,077648 | -1,432 |  |
| EVI2A | 0,891 | 7,816 | 3,245 | 0,00211 | 0,077648 | -1,435 |  |
| FYB | 0,986 | 7,184 | 3,245 | 0,00211 | 0,077648 | -1,435 |  |
| RBP2 | -1,054 | 6,662 | -3,245 | 0,00211 | 0,077648 | -1,436 |  |
| CD163 | 1,230 | 10,133 | 3,236 | 0,002163 | 0,078384 | -1,457 |  |
| ITGBL1 | 1,158 | 7,858 | 3,234 | 0,002175 | 0,078384 | -1,462 |  |
| PIGZ | -1,057 | 11,668 | -3,234 | 0,002178 | 0,078384 | -1,463 |  |
| CRLS1 | -0,722 | 11,705 | -3,231 | 0,002197 | 0,078504 | -1,470 |  |
| LOC389332 | -1,484 | 8,698 | -3,223 | 0,002249 | 0,079807 | -1,491 |  |
| LCP2 | 0,863 | 7,924 | 3,215 | 0,002297 | 0,080913 | -1,509 |  |
| LILRB3 | 0,765 | 8,504 | 3,208 | 0,002346 | 0,082078 | -1,528 |  |
| PRUNE2 | -1,171 | 8,037 | -3,202 | 0,002387 | 0,082913 | -1,543 |  |
| FCGR3A | 1,149 | 9,647 | 3,197 | 0,00242 | 0,083482 | -1,555 |  |
| GNB4 | 0,975 | 8,568 | 3,193 | 0,002449 | 0,083876 | -1,565 |  |
| CXCR4 | 1,089 | 11,594 | 3,188 | 0,002488 | 0,084626 | -1,578 |  |
| CD48 | 0,725 | 6,214 | 3,169 | 0,002623 | 0,087354 | -1,624 |  |
| SHD | -0,690 | 9,406 | -3,167 | 0,002641 | 0,087354 | -1,630 |  |
| LOC388242 | 0,797 | 6,958 | 3,161 | 0,002686 | 0,087354 | -1,645 |  |
| CTTNBP2 | -1,148 | 8,196 | -3,160 | 0,002688 | 0,087354 | -1,646 |  |
| COMP | 1,523 | 8,509 | 3,160 | 0,002689 | 0,087354 | -1,646 |  |
| NR3C1 | 0,761 | 7,524 | 3,157 | 0,002714 | 0,087354 | -1,654 |  |
| EDN2 | -0,954 | 6,530 | -3,157 | 0,002717 | 0,087354 | -1,655 |  |
| EVI2B | 0,828 | 7,729 | 3,154 | 0,002738 | 0,087354 | -1,662 |  |
| SLCO4A1 | -0,854 | 10,572 | -3,152 | 0,002751 | 0,087354 | -1,666 |  |
| PLXDC2 | 0,908 | 8,510 | 3,152 | 0,002751 | 0,087354 | -1,666 |  |
| LILRB5 | 0,960 | 7,023 | 3,151 | 0,002761 | 0,087354 | -1,669 |  |
| LOC100507930 | -1,110 | 6,617 | -3,149 | 0,00278 | 0,087395 | -1,675 |  |
| IGSF21 | 0,921 | 6,742 | 3,144 | 0,002815 | 0,087945 | -1,686 |  |
| IGANRP | 0,782 | 6,444 | 3,142 | 0,002833 | 0,087961 | -1,691 |  |
| FPR3 | 1,021 | 8,854 | 3,139 | 0,002857 | 0,088057 | -1,698 |  |
| LILRB2 | 0,946 | 7,801 | 3,135 | 0,002893 | 0,088057 | -1,709 |  |
| LOC100133306 | -0,708 | 7,003 | -3,134 | 0,002894 | 0,088057 | -1,710 |  |
| GALNT14 | 1,074 | 6,588 | 3,132 | 0,002915 | 0,088057 | -1,716 |  |
| MOXD1 | 1,331 | 7,474 | 3,131 | 0,002925 | 0,088057 | -1,719 |  |
| HSD11B2 | -1,163 | 10,850 | -3,127 | 0,00296 | 0,088573 | -1,729 |  |
| TIMP2 | 1,012 | 12,409 | 3,123 | 0,00299 | 0,088814 | -1,738 |  |
| GLIPR2 | 0,857 | 8,682 | 3,121 | 0,00301 | 0,088814 | -1,744 |  |
| LOC645638 | 1,015 | 7,865 | 3,118 | 0,003035 | 0,088814 | -1,751 |  |
| TMEM119 | 1,161 | 6,982 | 3,117 | 0,00304 | 0,088814 | -1,752 |  |
| ITGAM | 0,730 | 7,016 | 3,114 | 0,003067 | 0,088961 | -1,760 |  |
| GMFG | 0,947 | 10,822 | 3,111 | 0,003089 | 0,088961 | -1,766 |  |
| GGTA1P | 0,703 | 6,404 | 3,110 | 0,003099 | 0,088961 | -1,769 |  |
| MGC4294 | 0,693 | 6,290 | 3,106 | 0,003133 | 0,089437 | -1,778 |  |
| SAMSN1 | 0,833 | 6,772 | 3,103 | 0,003161 | 0,089729 | -1,786 |  |
| LILRB1 | 0,784 | 7,075 | 3,096 | 0,003222 | 0,09052 | -1,803 |  |
| TSC22D3 | 0,975 | 11,994 | 3,096 | 0,003226 | 0,09052 | -1,804 |  |
| BEND7 | -0,803 | 8,047 | -3,092 | 0,003264 | 0,09052 | -1,814 |  |
| DSE | 0,840 | 9,712 | 3,090 | 0,003283 | 0,09052 | -1,819 |  |
| HCST | 0,893 | 10,045 | 3,088 | 0,0033 | 0,09052 | -1,823 |  |
| LY96 | 0,999 | 8,670 | 3,088 | 0,003303 | 0,09052 | -1,824 |  |
| GFPT2 | 1,053 | 7,267 | 3,086 | 0,003317 | 0,09052 | -1,828 |  |
| ISLR | 1,171 | 10,059 | 3,080 | 0,003376 | 0,09162 | -1,843 |  |
| MYO1F | 0,789 | 9,211 | 3,070 | 0,003469 | 0,09318 | -1,866 |  |
| VAX2 | 0,863 | 7,390 | 3,070 | 0,003471 | 0,09318 | -1,867 |  |
| TNFAIP8L2 | 0,721 | 6,955 | 3,066 | 0,003512 | 0,09378 | -1,877 |  |
| ADAP2 | 0,856 | 9,876 | 3,060 | 0,003574 | 0,094932 | -1,892 |  |
| MFSD7 | 0,751 | 8,157 | 3,053 | 0,003638 | 0,096124 | -1,908 |  |
| WASF3 | 1,268 | 6,369 | 3,051 | 0,003666 | 0,096307 | -1,914 |  |
| IFIT1 | 1,405 | 8,664 | 3,049 | 0,003684 | 0,096307 | -1,918 |  |
| F12 | -1,254 | 11,821 | -3,044 | 0,003734 | 0,096641 | -1,930 |  |
| IKZF1 | 0,694 | 7,309 | 3,044 | 0,003736 | 0,096641 | -1,930 |  |
| C19orf45 | -0,797 | 7,427 | -3,041 | 0,003764 | 0,096875 | -1,937 |  |
| LIN28B | 0,768 | 5,041 | 3,035 | 0,003826 | 0,09768 | -1,951 |  |
| PRRX1 | 1,152 | 7,601 | 3,031 | 0,003867 | 0,09768 | -1,960 |  |
| CHIT1 | 0,745 | 6,996 | 3,028 | 0,003902 | 0,09768 | -1,968 |  |
| C20orf46 | -1,133 | 8,602 | -3,027 | 0,003915 | 0,09768 | -1,971 |  |
| FGR | 0,955 | 9,826 | 3,026 | 0,003923 | 0,09768 | -1,973 |  |
| SLFN11 | 0,855 | 7,883 | 3,025 | 0,003936 | 0,09768 | -1,975 |  |
| GABRA2 | -0,924 | 6,141 | -3,025 | 0,003939 | 0,09768 | -1,976 |  |
| CCR5 | 0,914 | 8,661 | 3,023 | 0,003956 | 0,09768 | -1,980 |  |
| FLI1 | 0,705 | 7,324 | 3,022 | 0,003972 | 0,09768 | -1,983 |  |
| FAP | 1,028 | 9,930 | 3,014 | 0,004059 | 0,099311 | -2,002 |  |
| IFI44L | 1,477 | 7,591 | 3,010 | 0,004108 | 0,099452 | -2,012 |  |
| AKT3 | 0,746 | 7,100 | 3,008 | 0,004127 | 0,099452 | -2,016 |  |
| ADCY7 | 0,685 | 7,725 | 3,007 | 0,004135 | 0,099452 | -2,018 |  |
| TMEM130 | 0,806 | 5,770 | 3,005 | 0,004163 | 0,099452 | -2,024 |  |
| C6orf145 | 0,762 | 6,809 | 3,005 | 0,004165 | 0,099452 | -2,024 |  |

| **Data_ set 12** |  |  |  |  |  |  |  |
| --- | --- | --- | --- | --- | --- | --- | --- |
| Ingenuity Pathway Analysis Summary for 208 genes differentially expressed between NACT treated (n=15) versus non-treated (n=29) metastatic samples. Of 208 genes, 191 were eligble for IPA core analysis. | | | | | | | |
|  |  |  |  |  |  |  |  |
| *Canonical Pathway Analysis^1^* |  |  |  |  |  |  |  |
| **Top Canonical Pathways** | **p-value** | **z-score** | **molecules** | | | | |
| Natural Killer Cell Signaling | 7,12E-06 | NaN | LAIR1,TYROBP,PLCG2,AKT3,HCST,LILRB1,FCGR3A/FCGR3B,LCP2 | | | | |
| phagosome formation | 7,12E-06 | NaN | TLR2,PLCB4,MSR1,PLCG2,TLR1,TLR7,FCGR3A/FCGR3B,FCGR1B | | | | |
| TREM1 Signaling | 6,33E-05 | 2 449 | TLR2,TYROBP,PLCG2,TLR1,TLR7,AKT3 | | | | |
| Dendritic Cell Maturation | 2,01E-04 | 2 121 | TLR2,PLCB4,TYROBP,PLCG2,TREM2,AKT3,FCGR3A/FCGR3B,FCGR1B | | | | |
| Role of Macrophages, Fibroblasts and Endothelial Cells in Rheumatoid Arthritis | 3,92E-04 | NaN | TLR2,SFRP4,FZD10,PLCB4,PLCG2,TLR1,TLR7,AKT3,FCGR3A/FCGR3B,TNFSF13B | | | | |
| Role of NFAT in Regulation of the Immune Response | 9,33E-04 | 1 633 | GNB4,PLCB4,PLCG2,AKT3,FCGR3A/FCGR3B,LCP2,FCGR1B | | | | |
| Role of Pattern Recognition Receptors in Recognition of Bacteria and Viruses | 9,73E-04 | 2 449 | TLR2,PLCG2,TLR1,TLR7,C1QA,C1QB | | | | |
| Production of Nitric Oxide and Reactive Oxygen Species in Macrophages | 1,34E-03 | 2 449 | TLR2,PPP1R14C,PLCG2,NCF2,CYBB,AKT3,SPI1 | | | | |
| Hepatic Fibrosis / Hepatic Stellate Cell Activation | 1,43E-03 | NaN | MYL9,COL8A2,LY96,CCR5,COL4A5,IL10RA,TIMP2 | | | | |
| FcÎ³ Receptor-mediated Phagocytosis in Macrophages and Monocytes | 1,62E-03 | 2 236 | AKT3,FYB,FCGR3A/FCGR3B,FGR,LCP2 | | | | |
|  |  |  |  |  |  |  |  |
|  |  |  |  |  |  |  |  |
| *Functional Analysis of gene subset^2^* |  |  |  |  |  |  |  |
| **Category** | **Functions Annotation** | **p-value** | **Predicted Activation State** | **Activation z-score** | **# molecules** | **molecules** | |
| Cellular Movement | migration of cells | 3,92E-16 | Increased | 3 026 | 64 | AKT3,ALOX15B,ALOX5AP,CCDC88A,CCR5,CD48,CMKLR1,CSF1R,CXCR4,CYBB,DOCK10,DSE,EDN2,EHF,EVL,F12,FAP,FCGR3A/FCGR3B,FERMT1,FES,FGR,FYB,GLIPR2,HAVCR2,HCLS1,HOXB2,IGFBP6,IL10RA,ITGAM,ITGBL1,LCP1,LCP2,LEFTY1,LILRB3,LIN28B,LIPA,LY96,MS4A4A,MSR1,MYO1F,NCF2,NEXN,PECAM1,PIGR,PLA2G7,PLCG2,PRRX1,RGS1,SFRP4,SGK1,SLC1A3,SPARCL1,SPI1,TIMP2,TLR2,TLR7,TNFAIP8L2,TNFSF13B,TREM2,TRPV2,TSC22D3,TYROBP,VCAN,WASF3 | |
| Cardiovascular Disease | peripheral arterial disease | 6,73E-16 |  |  | 22 | ADAP2,CD163,CECR1,CXCR4,EVI2A,EVI2B,FCGR3A/FCGR3B,FYB,HCLS1,KCTD12,LAIR1,LAPTM5,LCP1,LILRB2,MAFB,MSR1,NR3C1,SAMSN1,SGK1,SLA,TLR7,VSIG4 | |
| Cellular Movement | cell movement | 1,06E-14 | Increased | 2 838 | 66 | AKT3,ALOX15B,ALOX5AP,CCDC88A,CCR5,CD48,CMKLR1,CSF1R,CXCR4,CYBB,DOCK10,DSE,EDN2,EHF,EVL,F12,FAP,FCGR3A/FCGR3B,FERMT1,FES,FGR,FYB,GLIPR2,HAVCR2,HCLS1,HOXB2,IGFBP6,IL10RA,ITGAM,ITGBL1,LCP1,LCP2,LEFTY1,LILRB3,LIN28B,LIPA,LY96,MS4A4A,MSR1,MYO1F,NCF2,NEXN,NR3C1,PECAM1,PIGR,PLA2G7,PLCG2,PRRX1,RGS1,SFRP4,SGK1,SLC1A3,SPARCL1,SPI1,TFF3,TIMP2,TLR2,TLR7,TNFAIP8L2,TNFSF13B,TREM2,TRPV2,TSC22D3,TYROBP,VCAN,WASF3 | |
| Immunological Disease | systemic autoimmune syndrome | 1,47E-14 |  |  | 47 | ALOX5AP,C1orf162,C1QA,CCR5,CD52,CD84,CECR1,COL4A5,COMP,CXCR4,CYBB,EVI2A,FCGR1B,FCGR3A/FCGR3B,FES,GABRA2,GLIPR2,GPNMB,HAVCR2,HCLS1,HCST,IFIT1,IKZF1,IL10RA,ITGAM,LCP1,LILRB3,LST1,LY86,MAFB,MS4A6A,MS4A7,MYO1F,NCF2,NR3C1,PECAM1,PLEK,PRUNE2,RGS1,SGK1,TLR1,TLR2,TLR7,TNFSF13B,TPM2,TYROBP,VSIG4 | |
| Cellular Function and Maintenance | function of leukocytes | 3,09E-14 |  |  | 31 | BCAT1,CCR5,CD84,CMKLR1,CSF1R,CXCR4,CYBB,FES,FGR,FYB,GPNMB,HCLS1,HCST,IL10RA,ITGAM,LCP1,LILRB3,LY96,MSR1,PECAM1,PIGR,PLCG2,RGS1,SAMSN1,SASH3,SLA,TLR1,TLR2,TLR7,TNFAIP8L2,TYROBP | |
| Cellular Function and Maintenance | function of blood cells | 6,88E-14 |  |  | 32 | BCAT1,CCR5,CD84,CMKLR1,CSF1R,CXCR4,CYBB,FES,FGR,FYB,GPNMB,HCLS1,HCST,IL10RA,ITGAM,LCP1,LILRB3,LY96,MSR1,PECAM1,PIGR,PLCG2,RGS1,SAMSN1,SASH3,SLA,TLR1,TLR2,TLR7,TNFAIP8L2,TYROBP,VSIG4 | |
| Cardiovascular Disease | peripheral vascular disease | 9,24E-14 |  |  | 27 | ADAP2,ALOX5AP,CD163,CECR1,CXCR4,CYBB,EVI2A,EVI2B,FCGR3A/FCGR3B,FYB,GABRA2,HCLS1,KCTD12,LAIR1,LAPTM5,LCP1,LILRB2,LY96,MAFB,MSR1,NR3C1,PLA2G7,SAMSN1,SGK1,SLA,TLR7,VSIG4 | |
| Cellular Movement, Immune Cell Trafficking | leukocyte migration | 2,67E-13 | Increased | 2 533 | 38 | ALOX5AP,CCDC88A,CCR5,CD48,CMKLR1,CSF1R,CXCR4,CYBB,EDN2,EVL,FCGR3A/FCGR3B,FGR,FYB,HCLS1,IL10RA,ITGAM,LCP1,LCP2,LILRB3,LIPA,LY96,MS4A4A,MSR1,MYO1F,PECAM1,PIGR,PLA2G7,PLCG2,RGS1,SPI1,TIMP2,TLR2,TLR7,TNFAIP8L2,TNFSF13B,TREM2,TRPV2,TYROBP | |
| Cellular Movement, Hematological System Development and Function, Immune Cell Trafficking | cell movement of leukocytes | 6,35E-13 | Increased | 2 099 | 35 | ALOX5AP,CCDC88A,CCR5,CD48,CMKLR1,CSF1R,CXCR4,CYBB,EDN2,FCGR3A/FCGR3B,FGR,FYB,HCLS1,IL10RA,ITGAM,LCP1,LCP2,LILRB3,LIPA,MS4A4A,MSR1,MYO1F,PECAM1,PIGR,PLA2G7,PLCG2,RGS1,SPI1,TIMP2,TLR2,TNFAIP8L2,TNFSF13B,TREM2,TRPV2,TYROBP | |
| Hematological System Development and Function, Tissue Morphology | quantity of leukocytes | 7,35E-12 |  | 1 930 | 39 | AKT3,C1QA,CCR5,CD48,CD84,CMKLR1,CSF1R,CXCR4,CYBB,FES,FLI1,FYB,HAVCR2,HCLS1,HCST,IKZF1,IL10RA,ITGAM,KLHL6,LAIR1,LCP2,LILRB3,MFAP5,NR3C1,PECAM1,PIGR,PLCG2,RGS1,SAMSN1,SASH3,SLA,SPI1,TIMP2,TLR2,TNFAIP8L2,TNFSF13B,TREM2,TYROBP,VSIG4 | |
| Infectious Diseases | Bacterial Infections | 1,05E-11 | Decreased | -2 710 | 25 | ADCY7,ALOX5AP,C1QA,C1QB,CCR5,CHIT1,CMKLR1,CYBB,FCGR1B,FCGR3A/FCGR3B,FES,FGR,ITGAM,LCP1,LILRB3,LILRB5,LY86,LY96,MSR1,NR3C1,PIGR,TLR1,TLR2,TRPV2,VSIG4 | |
| Cellular Development, Cellular Growth and Proliferation, Hematological System Development and Function | proliferation of immune cells | 1,24E-11 |  | 0,520 | 34 | CCR5,CD48,CD84,CSF1R,CXCR4,FLI1,FYB,HAVCR2,HCLS1,IKZF1,IL10RA,ITGAM,LAIR1,LAPTM5,LCP1,LCP2,LILRB1,LILRB2,LILRB3,LST1,LY86,LY96,NR3C1,PECAM1,PLCG2,SAMSN1,SASH3,SPI1,TLR2,TLR7,TNFSF13B,TSC22D3,TYROBP,VSIG4 | |
| Metabolic Disease | glucose metabolism disorder | 1,35E-11 |  | 0,094 | 46 | AKT3,ALOX5AP,C1orf162,C1QA,CCR5,CD52,CD84,CMKLR1,COL4A5,COL8A2,CXCR4,CYBB,EVI2A,GABRA2,GLIPR2,GPNMB,HAVCR2,HCLS1,HCST,IFIT1,IL10RA,ITGAM,LCP1,LILRB1,LILRB3,LIPA,LST1,LY86,MS4A6A,MSR1,MYO1F,NCF2,NR3C1,PCK1,PECAM1,PLCG2,PLEK,PRUNE2,RGS1,TLR2,TLR7,TNFSF13B,TSC22D3,TSPAN8,TYROBP,UCHL1 | |
| Cellular Development, Cellular Growth and Proliferation | proliferation of blood cells | 1,40E-11 |  | 0,733 | 35 | CCR5,CD163,CD48,CD84,CSF1R,CXCR4,FLI1,FYB,HAVCR2,HCLS1,IKZF1,IL10RA,ITGAM,LAIR1,LAPTM5,LCP1,LCP2,LILRB1,LILRB2,LILRB3,LST1,LY86,LY96,NR3C1,PECAM1,PLCG2,SAMSN1,SASH3,SPI1,TLR2,TLR7,TNFSF13B,TSC22D3,TYROBP,VSIG4 | |
| Hematological System Development and Function, Tissue Morphology | quantity of mononuclear leukocytes | 1,73E-11 | Increased | 2 248 | 34 | AKT3,CCR5,CD48,CD84,CMKLR1,CSF1R,CXCR4,FES,FLI1,FYB,HCLS1,HCST,IKZF1,IL10RA,ITGAM,KLHL6,LAIR1,LCP2,LILRB3,MFAP5,NR3C1,PECAM1,PIGR,PLCG2,RGS1,SAMSN1,SASH3,SLA,SPI1,TLR2,TNFAIP8L2,TNFSF13B,TYROBP,VSIG4 | |
|  |  |  |  |  |  |  |  |
|  |  |  |  |  |  |  |  |
| *Upstream Analysis^3^* |  |  |  |  |  |  |  |
| **Upstream Regulator** | **Exp Log Ratio** | **Molecule Type** | **Predicted Activation State** | **Activation z-score** | **p-value of overlap** | **Target molecules in dataset** | |
| IFNG |  | cytokine | Activated | 3 727 | 8,41E-11 | C1QA,C1QB,CCR5,CD163,CECR1,CH25H,CXCR4,CYBB,FCGR1B,GNB4,IFI44L,IFIT1,IL10RA,ITGAM,KCTD12,NCF2,PCK1,PIGR,PPP1R1B,SLC15A3,SPI1,THEMIS2,TLR1,TLR2,TNFSF13B,TYROBP | |
| TGM2 |  | enzyme | Activated | 3 162 | 1,62E-05 | DCSTAMP,FERMT1,IFIT1,IL10RA,ITGAM,LILRB2,MAFB,NCF2,TYROBP,VSIG4 | |
| Hbb-b2 |  | other | Activated | 2 236 | 1,95E-05 | C1QA,CCR5,IL10RA,LY86,TLR2 | |
| CSF2 |  | cytokine | Activated | 2 234 | 8,05E-04 | ACP5,CSF1R,CYBB,ITGAM,LCP1,LY96,SPI1,TLR2 | |
| Interferon alpha |  | group | Activated | 2 200 | 8,42E-03 | IFIT1,TLR1,TLR2,TLR7,TNFSF13B | |
| HRG |  | other | Activated | 2 000 | 1,01E-05 | C1QA,C1QB,CXCR4,FCGR3A/FCGR3B | |
| SPI1 | 0,917 | transcription regulator |  | 1 982 | 2,06E-08 | CHIT1,CSF1R,CYBB,DCSTAMP,FES,IKZF1,ITGAM,LILRB3,NCF2,SPI1,TLR2 | |
| IL10 |  | cytokine |  | 1 927 | 3,74E-02 | CD163,FCGR3A/FCGR3B,LILRB2,VCAN | |
| MGEA5 |  | enzyme |  | 1 897 | 6,18E-04 | ALOX5AP,C1QA,C1QB,CCR5,CD48,CH25H,CSF1R,FZD10,GABRA2,TIMP2 | |
| STAT3 |  | transcription regulator |  | 1 794 | 5,14E-03 | CCDC88A,IFIT1,ITGAM,LILRB2,PCK1,SGK1,SLC1A3,VCAN | |
| APOE |  | transporter |  | -1 767 | 4,63E-04 | CCR5,CYBB,IGFBP6,IL10RA,ITGAM,LIPA,MSR1,NCF2 | |
| miR-155-5p (miRNAs w/seed UAAUGCU) |  | mature microrna |  | -1 982 | 2,73E-04 | CSF1R,MAFB,SLA,SPI1 | |
| PKD1 |  | ion channel | Inhibited | -2 000 | 5,65E-02 | ADCY7,ARHGAP10,BCAT1,PCK1 | |
| INSIG1 |  | other | Inhibited | -2 236 | 1,08E-03 | ALOX5AP,CCR5,CXCR4,LIPA,PLA2G7 | |
| IL10RA | 1 255 | transmembrane receptor | Inhibited | -2 646 | 1,23E-02 | AKT3,DCSTAMP,EHF,FERMT1,PCK1,SLAMF8,TLR2 | |
|  |  |  |  |  |  |  |  |
|  |  |  |  |  |  |  |  |
| ^1^Canonical pathway analysis identified from the Ingenuity Knowledge base that were most significant to the gene set. | | |  |  |  |  |  |
| ^2^Top ranked biological functions that were most significant to the genes eligible for analysis using a right tailed Fisher's exact test. | | | |  |  |  |  |
| ^3^Upstream regulator analysis based on the 191 genes using Ingenuity Knowledge base. Top ranked regulators according to activation/inhibition z-score are displayed. The z-score is based on relationships between experimentally observed gene expression and function annotation data, as derived from the information compiled in the Ingenuity Knowledge Base. These relationships are associated with a direction of change that is either activating (z-score ≥ 2) or inhibiting (z-score ≤ -2). For activated upstream regulators predicted in this analysis, the top 15 regulators are displayed. | | | | | | | |
